# DeepAden: an explainable machine learning method for predicting the substrate specificity of nonribosomal peptide synthetases

**DOI:** 10.1093/nar/gkag656

**Published:** 2026-07-06

**Authors:** Jiaquan Huang, Liangjun Ge, Yaxin Wu, Yi Tian, Jun Wu, Qiandi Gao, Pan Li, Song Meng, Heqian Zhang, Zhiwei Qin

**Affiliations:** Center for Biological Science and Technology, Advanced Institute of Natural Sciences, Beijing Normal University, Zhuhai, Guangdong 519087, People’s Republic of China; Center for Biological Science and Technology, Advanced Institute of Natural Sciences, Beijing Normal University, Zhuhai, Guangdong 519087, People’s Republic of China; Center for Biological Science and Technology, Advanced Institute of Natural Sciences, Beijing Normal University, Zhuhai, Guangdong 519087, People’s Republic of China; Center for Biological Science and Technology, Advanced Institute of Natural Sciences, Beijing Normal University, Zhuhai, Guangdong 519087, People’s Republic of China; Center for Biological Science and Technology, Advanced Institute of Natural Sciences, Beijing Normal University, Zhuhai, Guangdong 519087, People’s Republic of China; Center for Biological Science and Technology, Advanced Institute of Natural Sciences, Beijing Normal University, Zhuhai, Guangdong 519087, People’s Republic of China; Center for Biological Science and Technology, Advanced Institute of Natural Sciences, Beijing Normal University, Zhuhai, Guangdong 519087, People’s Republic of China; State Key Laboratory of Drug Research & Natural Products Research Center, Shanghai Institute of Materia Medica, Chinese Academy of Sciences, Shanghai 201203, People’s Republic of China; University of Chinese Academy of Sciences, Beijing 100049, People’s Republic of China; Zhongshan Institute for Drug Discovery, Shanghai Institute of Materia Medica, Chinese Academy of Sciences, Zhongshan 528400, People’s Republic of China; Center for Biological Science and Technology, Advanced Institute of Natural Sciences, Beijing Normal University, Zhuhai, Guangdong 519087, People’s Republic of China; Center for Biological Science and Technology, Advanced Institute of Natural Sciences, Beijing Normal University, Zhuhai, Guangdong 519087, People’s Republic of China

## Abstract

Microbial nonribosomal peptides (NRPs) exhibit remarkable structural diversity and are important sources of lead compounds for clinical drug development. The biosynthesis of NRPs relies on nonribosomal peptide synthetases (NRPSs), in which adenylation (A) domains define the core structure by selectively recognizing and activating amino acid substrates. Accurately predicting the substrate specificities of A-domains is thus essential for understanding the core structural and biosynthetic logic of NRPs. Here, we present DeepAden, a two-stage deep learning model. In the first stage, a graph attention network (GAT)-based model localizes 27-residue binding pockets within 6 Å of bound substrates and converts these into pocket representations. In the second stage, pocket representations are encoded alongside substrate information using pretrained language models and aligned using contrastive learning. We also applied a SHapley Additive exPlanations (SHAP)-guided data augmentation strategy to mitigate class imbalance, particularly focusing on nonproteinogenic substrates. DeepAden achieved competitive performance compared with state-of-the-art tools on a benchmark dataset and facilitated the annotation of two *Streptomyces* NRPS gene clusters by providing supportive A-domain substrate-specificity predictions. DeepAden provides a practical approach for pocket localization and substrate prediction and may facilitate the discovery and characterization of new NRP natural products. The DeepAden web server is available at https://deepnp.site/.

## Graphical Abstract

**Graphical Abstract:**
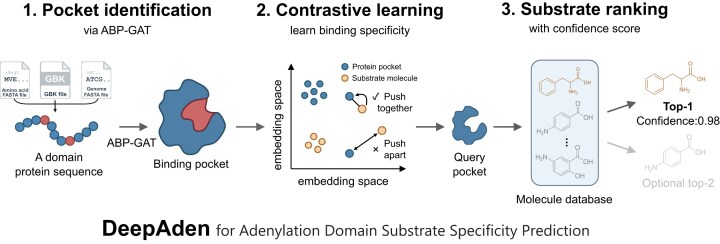

## Introduction

Nonribosomal peptides (NRPs) are naturally occurring biopolymers with diverse structures and functions, acting as antibiotics, siderophores, toxins, and immunosuppressants [1]. Assembly of their peptide backbones is mediated by modular nonribosomal peptide synthetases (NRPSs), which use proteinogenic or nonproteinogenic amino acids as monomeric building blocks [2]. NRPSs are minimally composed of three core domains: an adenylation (A) domain, which is responsible for substrate selection and activation; a peptidyl carrier protein (PCP) domain, which tethers activated amino acids via its phosphopantetheinyl prosthetic group; and a condensation (C) domain, which catalyzes the peptide bond formation procedure to elongate the chain [3]. In principle, the sequential arrangement of A-domains within biosynthetic gene clusters (BGCs) determines the core peptide sequence. Therefore, understanding their specificities is critical for predicting peptide structures [4, 5].

In 1997, Stachelhaus *et al*. reported the first crystal structure of an NRPS adenylation domain in complex with its substrate phenylalanine (GrsA-PheA, PDB ID: 1AMU) [6]. Based on this structural insight, they identified 10 key amino acid residues (10-AA) within the active site that interact directly with the substrate and proposed that these specificity-conferring codes (SCCs) could be used to predict A-domain substrate specificity. Building on this concept, Rausch *et al*. introduced an extended A-domain binding pocket (ABP), consisting of 34 amino acid residues (34-AA) located within an 8 Å radius of the substrate [7, 8]. The SCC and ABP frameworks have since underpinned the development of numerous A-domain substrate prediction tools, employing diverse computational approaches such as sequence alignment, hidden Markov models (HMMs) [9], support vector machines (SVMs) [10], and random forests (RF) [11]. Such tools, including NRPSpredictor2 [12], SANDPUMA [13], AdenPredictor [14], and PARAS [15], have performed well in identifying proteinogenic substrates. However, since these residues were defined based on a single co-crystal structure from one species and substrate class, their generalizability across phylogenetically diverse A-domains remains limited. Moreover, the scarcity of nonproteinogenic substrate datasets has significantly hindered progress in substrate specificity prediction. Therefore, it is advantageous to redefine SCCs and ABP residues by incorporating a broader set of co-crystal structures representing diverse A-domains and substrates for more accurate and generalizable identification.

Current extraction of binding pocket residues in A-domains mainly relies on sequence alignment, but accuracy drops for domains distant from the GrsA-PheA structure or those recognizing non-proteinogenic substrates [3]. Additionally, key patterns are often unclear in 1D sequences but conserved in 3D structures [16, 17]. Incorporating structural information into computational models may improve the identification of SCC or ABP residues and, ultimately, substrate specificity prediction. Recent advances in deep learning have transformed computational biology by enabling the extraction of complex patterns from large-scale datasets, including implicit structural features [18–20]. Protein language models (PLMs) and chemical language models (CLMs), such as Evolutionary Scale Modeling 2 (ESM2) and MoLFormer, exemplify this progress, leveraging self-supervised learning to uncover evolutionary and structural patterns from vast protein sequence and chemical structure corpora [21, 22]. Transfer learning can further enhance these models by enabling the extraction of fine-grained structural and functional features, facilitating tasks such as protein function prediction and residue-level mutational impact analysis [20, 23, 24]. For instance, the ESM2-650M model has demonstrated accurate residue-residue contact prediction in both active and inactive peptides, which directly facilitates the engineering of antimicrobial peptides [20]. More recently, protein structures and the spatial relationships between residues have been effectively represented as graphs for analysis using graph neural networks (GNNs) [25]. By reframing binding pocket residue identification from a sequence-based to a graph-based problem, it becomes possible to harness the conserved spatial patterns characteristic of A-domains. This shift holds significant potential for improving the precision of ABP residue identification and advancing substrate-specificity prediction.

Another challenge in substrate specificity prediction lies in the probabilistic formulation of prediction models. Most existing tools rely on softmax-based multiclass classification, which treats substrate labels as mutually exclusive and redistributes probabilities across all candidate substrates. This coupling can distort confidence estimates for individual substrates. Accordingly, there is growing interest in alternative formulations that assign independent confidence scores to each candidate substrate. Recent progress in protein-ligand interaction prediction provides a useful conceptual and technical foundation for such developments. Features derived from PLMs and CLMs can be leveraged to construct high-dimensional representations of binding pockets and ligands, and contrastive learning has emerged as an effective strategy to align these representations in a shared embedding space [26]. In this framework, contrastive objectives pull together embeddings of true protein-ligand pairs while pushing apart mismatched pairs, thereby enhancing representation quality and enabling more accurate interaction scoring and ranking. Emerging alignment-free approaches, inspired by virtual screening, predict protein-substrate interactions directly from protein sequences and molecular descriptors such as SMILES strings. State-of-the-art models trained on binding-affinity datasets enable systematic identification of likely interaction pairs, while architectures that integrate PLMs and CLMs offer improved robustness and adaptability to unseen substrates [27]. In parallel, explainable artificial intelligence has become increasingly valuable for identifying informative features and rationalizing model behavior in complex biological prediction tasks [28]. SHapley Additive exPlanations (SHAP) offers a principled framework for quantifying feature contributions, while TreeSHAP [29] enables consistent and interpretable attribution analysis for tree-based models. Such approaches have proven valuable for revealing residue-level determinants underlying protein properties [30].

To address these limitations, we redefined 27 residues as A-domain binding pocket (27-AA ABP) derived from evolutionarily diverse pocket-substrate pairs, forming a compact, spatially coherent cavity that preserves critical contacts, reduces structural noise, and can be efficiently encoded by a graph attention network (GAT). Building on this, we developed DeepAden, a two-stage ensemble tool for A-domain substrate-specificity prediction, composed of three innovative modules: (i) a residual neural network [31] (ResNet)-based residue contact predictor that utilizes spatial constraints derived from PLM attention maps to capture the structural features of A-domains; (ii) a GAT-based ABP-prediction module (ABP-GAT) that combines residue-level features with probabilistic contact edges, enabling dynamic modeling of binding-pocket variation without reliance on predefined templates; and (iii) a contrastive-learning framework that uses ABP-ESM2 to encode the predicted ABPs and MoLFormer to encode substrates, thereby aligning their cross-modal representations while addressing class imbalance through SHAP [32]-guided data augmentation. In real-world accuracy comparisons, DeepAden achieves performance that is comparable to or better than existing A-domain substrate prediction tools, particularly under low-homology and nonproteinogenic settings, in the examples presented in this work. Additionally, we applied DeepAden to predict A-domain substrate specificities within orphan NRPS BGCs and to support their linkage to candidate metabolites in representative case studies. Our results indicate that DeepAden can serve as a useful tool for A-domain substrate specificity prediction and for supporting NRPS BGC annotation in natural product studies.

## Materials and methods

### Data collection and processing

#### A-domain sequence data with known labels

A-domain sequences with known labels were collected from three sources: the MIBiG database (v3.1) [33, 34] containing experimentally validated or structurally supported data points (1627 sequences); the PARAS dataset [15] (3257 sequences); and the NRPStransformer dataset [35] (4430 sequences). The raw data were processed through the following sequential steps. (i) Sequence redundancy removal was performed using CD-HIT (version 4.8.1) [36] with a 95% sequence identity threshold. (ii) Sequences lacking substrate labels or containing “branch” annotations were excluded. (iii) Synonymous labels were standardized, and erroneous substrate annotations were corrected through literature curation. (iv) To ensure compatibility with the molecular language model MoLFormer [22], which does not distinguish molecular stereoisomers, all substrate SMILES representations were canonicalized using RDKit (http://www.rdkit.org). (v) Since many data points possessed multiple substrate labels potentially arising from post-A-domain modifications by tailoring enzymes (e.g. methyltransferases, hydroxylases, and halogenases) within biosynthetic gene clusters (BGCs), we excluded A-domain sequences with multi-substrate labels likely attributable to such modifications. The remaining multi-substrate entries were merged with single-substrate data, ultimately yielding 4545 A-domain sequences spanning 223 substrate labels for training the substrate prediction model (Dataset S1, S2). To evaluate model generalization, we used an independent benchmark dataset from real-world sources, explicitly ensuring that no sequences overlapped with the training data. This benchmark dataset comprises 201 A-domain sequences, including 155 entries obtained from the NRPStransformer benchmark dataset (not included in 4430 sequences dataset) and 46 entries collected from the MIBiG database (v4.0). These data were used exclusively for performance evaluation (Dataset S3).

#### A-domain sequence data with unknown labels

To identify bacterial-derived NRPS A-domain sequences for subsequent fine-tuning of the ABP-ESM model, we implemented a sequence collection pipeline. First, all bacterial genomes were retrieved from the Genome Taxonomy Database (GTDB Release 220) [37], and BGCs were predicted using antiSMASH (v7.0) [38]. Coding sequences encoding NRPs were then extracted, generating a large pool of putative NRP gene cluster sequences. A-domains within these sequences were isolated through sequence alignment against known A-domains described earlier with diamond (v2.1.11) [39], yielding 82 748 successfully aligned A-domain sequences. In parallel, we retrieved 152 502 A-domain sequences from the UniRef database (release-2024_06). Following merging and deduplication of these two datasets, 233 914 unique sequences were obtained. These sequences were subsequently validated through HMM-based matching against NRPS A-domain profiles (Pfam IDs: PF00501.29 and PF13193.7) using HMMER (v3.3.2). After stringent filtering and deduplication, we ultimately obtained 186 758 unlabeled A-domain sequences with an average length of 495 amino acids.

#### A-domain structure data and binding pocket annotation

We first collected 10 A-domain structures co-crystallized with their substrates as the initial structural set for binding pocket definition. For each A-domain-substrate co-crystal structure, residues located within 6 Å or 8 Å of the bound substrate were identified in PyMOL v3.1.0, generating structure-specific pocket residue sets under the two distance cutoffs. These residue sets were then mapped to a common sequence-structure alignment, and the union across all 10 structures was used to define two ABP sets: a 27-residue ABP for the 6 Å cutoff and a 49-residue ABP for the 8 Å cutoff. To enhance dataset diversity and model generalization capability, we expanded the initial collection by retrieving homologous structures based on similarity criteria: sequence identity >30% and root-mean-square deviation (RMSD) < 3 Å. Following quality assessment and redundancy removal, the final curated dataset comprised 78 A-domain structures (Dataset S4), which were subsequently utilized to train the residue contact prediction model and the GAT-based binding pocket prediction model.

For binding-pocket annotation of the 78 structures, GrsA (PDB: 1AMU) bound to the proteinogenic substrate Phe and DhbE (PDB: 1MD9) bound to the nonproteinogenic substrate Dhb were used as reference templates for both the 27-AA and 49-AA ABP definitions. For each query structure, structural similarity to the two templates was first assessed using Foldseek [40] with default parameters. The best-matching template was then selected according to the Foldseek alignment score, followed by structure-based alignment in PyMOL using the align command. The 27-AA or 49-AA pocket annotations were then transferred from the selected template to the query structure based on residue correspondence. The mapped residues were subsequently inspected manually, and misaligned or inconsistent assignments were corrected using FoldMason [41], yielding curated reference sets of ABP residues for the 78 A-domain structures.

### Construction of ResNet-based residue contact prediction model

#### Model architecture

We developed a deep-learning framework for predicting binary residue-residue contacts from protein sequences, utilizing evolutionary-scale information from protein language models [21]. Our architecture comprises two main components: (i) a pretrained protein language model serving as a feature extractor and (ii) a specialized residual projection histogram network for contact-map prediction.

We employed ESM2 as our backbone feature extractor. Specifically, we utilized the esm2_t33_650M_UR50D variant containing 33 transformer layers with 650 million parameters. For each input protein sequence *S* of length *L*, we obtained:


\begin{eqnarray*}
{\mathrm{E}},{\mathrm{\ A}} = {\mathrm{ESM}}(S),
\end{eqnarray*}

where$\ {\mathrm{E}}\in {{\mathbb{R}}^{L \times d}}$ represents the residue-wise embeddings (*d* = 1280), and ${\mathrm{A}}\in {{\mathbb{R}}^{33 \times 20 \times L \times L}}$ denotes the multi-head attention maps across all transformer layers and attention heads. These attention maps capture pairwise dependencies between residues at different abstraction levels.

To transform attention maps into contact predictions, we designed a residual projection histogram network. The attention maps from all layers and heads (${\mathrm{A}}$) are flattened along the layer dimension, resulting in ${\mathrm{A^{\prime}}}\in {{\mathbb{R}}^{660 \times L \times L}}$. A series of six residual blocks progressively reduce feature dimensions: 660→512→256→128→64→32→16 channels. Each residual block contains two convolutional layers with batch normalization and ReLU activations, with skip connections to mitigate vanishing gradients. A final 1 × 1 convolution produces logits ${\mathrm{O}}\in {{\mathbb{R}}^{L \times L}}$, which are symmetrized to ensure consistency:


\begin{eqnarray*}
{{{\mathrm{O}}}_{{\mathrm{sym}}}} = \frac{1}{2}({{\mathrm{O}} + {{{\mathrm{O}}}^ \top }}).
\end{eqnarray*}

The output is passed through a sigmoid function to obtain contact probabilities ${\mathrm{P}}\in {{[ {0,{\mathrm{\ }}1} ]}^{L \times L}}$.

We formulated contact prediction as a binary classification problem. Given ground truth contact matrix ${\mathrm{Y}}\in {{\{ {0,{\mathrm{\ }}1} \}}^{L \times L}}$ (where contacts are defined as residue pairs with C_β_-C_β_ distance < 8 Å), we minimized the binary cross-entropy loss (BCE loss).

#### Training

The 78 curated A-domain structures were used to generate residue contact maps based on C*_β_* atom distances with an 8 Å cutoff threshold. The dataset was randomly split into training and validation subsets at an 8:2 ratio. The model was trained using AdamW optimizer [42] with initial learning rate 1 × 10⁻³, weight decay 1 × 10⁻², and batch size 1. We employed a ReduceLROnPlateau scheduler that reduced the learning rate by a factor of 0.8 when the validation Matthews correlation coefficient (MCC) plateaued for 15 epochs, with a minimum learning rate of 1 × 10⁻⁶. Model performance was assessed using multiple metrics calculated per-protein and averaged:


\begin{eqnarray*}
{\mathrm{Accuracy\ }}({{\mathrm{ACC}}}) = \frac{{TP + TN}}{{TP + TN + FP + FN}}
\end{eqnarray*}

\begin{eqnarray*}
{\mathrm{Precision\ }}({{\mathrm{PRE}}}) = \frac{{TP}}{{TP + FP}}
\end{eqnarray*}

\begin{eqnarray*}
{\mathrm{Recall\ }}( {{\mathrm{REC}}}) = \frac{{TP}}{{TP + FN}}
\end{eqnarray*}

\begin{eqnarray*}
{\mathrm{F}}1 - {\mathrm{score}} = 2 \times \frac{{{\mathrm{PRE}} \times {\mathrm{REC}}}}{{{\mathrm{PRE}} + {\mathrm{REC}}}}
\end{eqnarray*}

\begin{eqnarray*}
&&{\mathrm{Matthews\ Correlation\ Coefficient\ }}({{\mathrm{MCC}}})\\&& = \frac{{TP \times TN - FP \times FN}}{{\sqrt {({TP + FP})({TP + FN})({TN + FP})({TN + FN})} }}.
\end{eqnarray*}

We implemented a dynamic threshold optimization procedure, evaluating thresholds from 0 to 1 in 0.05 increments to maximize MCC on the validation set. The best-performing model checkpoint was selected based on validation MCC. All experiments were conducted using PyTorch v2.1.0 with CUDA 12.0 acceleration on a single NVIDIA A100 GPU. The model parameters were initialized from pretrained ESM2 weights, with the convolutional layers initialized using Kaiming initialization [43].

### A GAT-based node classification model for A-domain binding pocket prediction

#### PLM-based graph construction

For a given 1D amino acid sequence with ${{N}_p}$ residues, DeepAden generated a 2D protein contact map graph with ${{N}_p}$ residue nodes using ESM2. The protein graph was characterized by ${{P}^{( 0 )}}$, $CM_j^i$ and $E_p^{( 0 )}$, representing the residue node features, the contact map matrix that connected the nodes, and the edge features describing these connections, respectively. A residue feature was computed as follows:


(1) \begin{eqnarray*}
{{P}^{(0)}} = {\mathrm{ML}}{{{\mathrm{P}}}_{{\mathrm{esm}}}}\left( {P_{{\mathrm{esm}}}^{(0)}} \right) + {\mathrm{ML}}{{{\mathrm{P}}}_{{\mathrm{pc}}}}\left( {P_{{\mathrm{pc}}}^{(0)}} \right),
\end{eqnarray*}

where ${{P}^{( 0 )}}\in {{\mathbb{R}}^{{{N}_p} \times 512}}$ represents the computed residue feature; $P_{{\mathrm{esm}}}^{( 0 )}\in {{\mathbb{R}}^{{{N}_p} \times 1280}}$ represents the residue-type feature obtained from the final layer of ESM2; and $P_{{\mathrm{pc}}}^{( 0 )}\in {{\mathbb{R}}^{{{N}_p} \times 18}}$ represents the physicochemical properties that encode the residue weights, pK values, the hydrophobicity status, and whether the residue is aliphatic, aromatic, neutral, or charged. The contact map matrix $CM_j^i$ was predicted via ResNet with 6 residual blocks on the ESM2 attention map, and the edge features $E_p^{( 0 )}$ were obtained from the predicted contact probabilities obtained from the ResNet output, where each element $CM_j^i$ indicates the probability of contact between residues *i* and *j*.

#### GAT-based binding pocket prediction model architecture and evaluation

The GAT model served as the foundational architecture for conducting residue-level classifications on protein graphs; this process specifically targeted the prediction of binding pocket residues. After constructing the graph, where each node is characterized by an initial feature ${{P}^{( 0 )}}$ and each edge is characterized by a feature $E_p^{( 0 )}$, a GAT was employed to embed the constructed graphs into fixed-size latent representations, typically those residues involved in the binding pocket. The core of the model was built upon four GATConv layers. In each layer, the model aggregated information from its neighboring nodes using an attention mechanism. Specifically, for the $l - th$ layer, the feature update for node *i* was defined as follow:


(2) \begin{eqnarray*}
{\mathrm{h}}_i^{(l + 1)} = {\mathrm{Dropout}}\left( {{\mathrm{\sigma }}\left( {\mathop \sum \limits_{j\in \mathcal{N}(i)} {\mathrm{\alpha }}_{ij}^{(l)}{{{\mathrm{W}}}^{(l)}}{\mathrm{h}}_j^{(l)}} \right)} \right),
\end{eqnarray*}

where ${\mathrm{h}}_i^{( l )}$ refers to the feature vector of node *i* at layer *l*, ${{{\mathrm{W}}}^{( l )}}$ is the learnable weight matrix at layer *l*, ${\mathrm{\alpha }}_{ij}^{( l )}$ denotes the attention coefficient between node *i* and its neighbor *j* (with${\mathrm{\ j}}\in {\mathrm{N}}( {\mathrm{i}} ))$, ${\mathrm{\sigma }}( \cdot )$ is an ELU activation function that is applied after each convolution except that in the final layer, and dropout is used to randomly zero out a fraction of the activations to mitigate overfitting. The final convolutional layer, referred to as conv4, produced logits for each node:


(3) \begin{eqnarray*}
{{z}_i} = {\mathrm{conv}}4\left( {h_i^{(3)}} \right),
\end{eqnarray*}

where ${{z}_i}$ represents the unnormalized score produced for each class. These logits were subsequently transformed into probabilities via the Softmax function during the loss computation. Owing to the limited amount of training data, we further incorporated data augmentation and consistency regularization. Specifically, Gaussian noise was added to the initial node embeddings to generate perturbed versions of the node features:


(4) \begin{eqnarray*}
\widetilde {{\mathrm{h}}_i^{(0)}} = {\mathrm{h}}_i^{(0)} + {\mathrm{\varepsilon }}\left( {{\mathrm{\varepsilon }}\sim \mathcal{N}\left( {0,{{{\mathrm{\sigma }}}^2}I} \right)} \right),
\end{eqnarray*}

where *σ* controls the standard deviation of the noise.

To further enhance the robustness of the initial multilayer perceptron (MLP) classifier, we employed a consistency regularization-based semisupervised framework, as used by Huang *et al*. [24]. The datasets consisting of A-domain structures with and without substrate information are described in the Supplementary File. The overall training loss is defined as follows:


(5) \begin{eqnarray*}
{{L}_{\textit{total}}} = {{L}_{CE}}\ + \ {\mathrm{\lambda }}{{L}_{\textit{cons}}},
\end{eqnarray*}

where λ is a hyperparameter that balances the contribution of the consistency regularization scheme relative to the cross-entropy loss. The detailed hyperparameter configurations are summarized in Supplementary Table S1. Models based on both the 27-AA and 49-AA ABP definitions were trained and compared within the same GAT framework. Evaluation was primarily performed using leave-one-out cross-validation, with each sample held out once for testing and the remaining samples used for training. Residue-level predictions from all held-out samples were aggregated to obtain the final performance estimates. Residue-level performance was quantified using the F1 score, and 95% bootstrap confidence intervals were estimated by bootstrap resampling of the aggregated held-out predictions. To further assess the recoverability of complete ABP residue sets, we evaluated ABP-set recovery on a curated set of 4545 A-domains. The ABP-set recovery rate was defined as the proportion of A-domains for which the complete ABP residue set could be assigned after post-processing, rather than the proportion of individual residues correctly predicted.

### LoRA fine-tuning of the ESM2 for A-domain binding pocket characterization

To characterize the 27-AA ABP of A-domains, we fine-tuned the ESM2 pretrained model (esm2_t33_650M_UR50D) using a masked language modeling task on a curated dataset of 191 303 ABP sequences (with and without substrate information), resulting in ABP-ESM2 (Supplementary File). The dataset was randomly split into training and validation sets with a 9:1 ratio. To enable multi-GPU distributed and mixed-precision training, we implemented the fine-tuning procedure in the PyTorch Lightning (v2.3.3) framework combined with the parameter-efficient tuning method LoRA [44]. We applied random masking (masking rate of 10%) to the ABP sequences for masked language modeling. The LoRA configuration was set as follows: rank (*r* = 16), scaling factor (*α* = 64), and dropout rate (0.1).

During training, we used the cross-entropy loss function computed only at masked positions for amino acid prediction and adopted the AdamW optimizer. The initial learning rate was dynamically adjusted based on validation performance using a plateau-based scheduler with a patience of 5 epochs and a decay factor of 0.5. Training was run for up to 500 epochs with early stopping based on validation loss, and the best-performing checkpoint was retained for subsequent analyses and applications. All training was conducted in data-parallel mode on NVIDIA A100 GPUs. The resulting ABP-ESM2 model is directly used to characterize the binding pocket sites of A-domains and to provide feature representations for downstream tasks.

### SHAP-guided functional residue recognition in A-domain binding pockets

#### A-domain binding pocket residue prediction and sequence analysis

The predicted ABP residues of 4545 A-domain were concatenated into continuous sequences and then aligned using MAFFT v7.310 [45]. An ABP phylogenetic tree was constructed from the multiple sequence alignment with IQ-TREE v3.0.1 [46] and visualized using iTOL [47]. To assess how well the phylogeny reflects substrate specificity, we cut the tree at a fixed height threshold (H = 2.0 substitutions) to obtain leaf clusters and compared these tree-derived clusters with known substrate labels using the adjusted Rand index (ARI) [48], where ARI = 1 indicates perfect agreement and ARI ≈ 0 is expected for random clustering. Within bacterial sequences, we additionally computed one-versus-rest per-label ARI for substrate labels represented by at least 2 sequences and summarized results for labels with N ≥ 20, which were visualized as vertical bar plots in Python.

#### TreeSHAP model construction process for functional and substrate-specific residue analysis

For the TreeSHAP-based analysis, we used five-fold cross-validation on the 4545 ABP sequences and performed random oversampling only within the training subset of each fold. For each substrate class with fewer than 10 training samples, minority-class sequences were randomly duplicated until at least 10 sequences were available, while the corresponding validation subset was left unchanged. This oversampled dataset was used only for the explainability analysis and did not affect the training or evaluation of the main predictive models. We then trained one-versus-rest random forest classifiers for each substrate class using 27-AA pocket features derived from ABP-ESM2, with five-fold cross-validation and hyperparameter optimization via GridSearchCV (Supplementary Table S1). For each fold, TreeSHAP was applied to the corresponding validation set to compute per-position feature contributions, and SHAP values from all folds were aggregated to obtain out-of-fold importance profiles for each substrate class. These TreeSHAP-based random forest models are used purely as post hoc interpretability tools and are not part of the primary prediction pipeline of DeepAden. The SHAP value for feature *i* of sample *x* belonging to class *c* is defined as follows:


(6) \begin{eqnarray*}
{{\phi}_i}(f,x,c) = \mathop \sum \limits_{\left( {S \subseteq N \setminus \left\{ i \right\}} \right)} \frac{{\left| S \right|!\left( {\left| N \right| - \left| S \right| - 1} \right)!}}{{\left| N \right|!}}\left[ {{{f}_c}\left( {S\cup\left\{ i \right\}} \right) - {{f}_c}(S)} \right],\\
\end{eqnarray*}

where *N* is the set of all features, *S* is a subset of the features excluding *i*, and ${{f}_c}$ is the expected prediction of class *c* when only the features in set *S* are known. In the one-versus-rest setting, ${{f}_c}$ denotes the output of the one–versus–rest random forest classifier corresponding to class *c*. We analyzed the SHAP values across different classes to identify substrate-specific residues. For each site *i*, we calculated the mean absolute SHAP value across all classes:


(7) \begin{eqnarray*}
{\mathrm{SiteImportanc}}{{{\mathrm{e}}}_i} = \frac{1}{{n \times C}}\mathop \sum \limits_{j = 1}^n \mathop \sum \limits_{c = 1}^C \left| {{\mathrm{\phi}_i}\left( {f,{{x}_j},c} \right)} \right|,
\end{eqnarray*}

where *C* is the number of classes and *n* is the number of samples. For specific classes of interest, we compared their SHAP profiles with those of other classes to identify discriminative residues:


(8) \begin{eqnarray*}
{\mathrm{ClassSpecificImportanc}}{{{\mathrm{e}}}_{i,c}} = \frac{1}{{\left| {{{X}_c}} \right|}}\mathop \sum \limits_{{{x}_j}\in {{X}_c}} {\mathrm{\phi}_i}\left( {f,{{x}_j},c} \right),
\end{eqnarray*}

where ${{X}_c}$ is the set of samples belonging to class *c*.

### Contrastive learning for pocket-substrate binding prediction

To model pocket-substrate interactions, we built upon the supervised contrastive learning framework introduced in DrugCLIP. We adapted this paradigm to the A-domain substrate prediction task by incorporating sequence-based pocket representations and SMILES-based molecular embeddings. The overall training objective and contrastive formulation followed the DrugCLIP framework, while several domain-specific modifications were introduced, including tailored feature encoders, SHAP-guided data augmentation, and KDE-based probability calibration.

#### Pocket and substrate featurization

For cross-modal learning, A-domain binding pockets and substrates were encoded using pretrained models. Pocket sequences were represented using ABP-ESM2. For a protein pocket with a length of *n*, the pocket encoder generated a pocket embedding ${{P}_{\textit{full}}}\in {{\mathbb{R}}^{n \times {{d}_p}}}$ (${{d}_p}$=256), which was mean-pooled along the length of the pocket, resulting in a vector $P\in {{\mathbb{R}}^{{{d}_p}}}$. Substrates were represented from canonical SMILES strings using the pretrained chemical language model called MoLFormer [22], yielding molecular embeddings $M\in {{\mathbb{R}}^{{{d}_m}}}$, ${{d}_m}$= 768. Both encoders were used as feature extractors and were kept frozen during contrastive training.

#### Projection and prediction

Given a pocket embedding $P\in {{\mathbb{R}}^{{{d}_p}}}$and a ligand embedding $M\in {{\mathbb{R}}^{{{d}_m}}}$, we projected them into a shared embedding space (${{P}^*},{{M}^*}\in {{\mathbb{R}}^d}$, *d*= 128) using two separate two-layer MLPs:


(9) \begin{eqnarray*}
{{{\mathrm{P}}}^{\mathrm{*}}} = {\mathrm{ML}}{{{\mathrm{P}}}_{{\mathrm{pro}}}}\left( P \right)
\end{eqnarray*}

(10) \begin{eqnarray*}
{{{\mathrm{M}}}^{\mathrm{*}}} = {\mathrm{ML}}{{{\mathrm{P}}}_{{\mathrm{mol}}}}\left( M \right),
\end{eqnarray*}

where $ML{{P}_{pro}}:{{\mathbb{R}}^{{{d}_p}}}\to {{\mathbb{R}}^d}$ and $ML{{P}_{mol}}:{{\mathbb{R}}^{{{d}_m}}}\to {{\mathbb{R}}^d}$ are two-layer perceptrons with rectified linear unit (ReLU) activation [49]. Given the latent embeddings ${{P}^*},{{M}^*}$in the shared space, we employed cosine similarity as a metric to measure their binding affinity $\hat{p}( {{{P}^*}{\mathrm{,}}{{M}^*}} )\ = \ cos( {{{P}^*}{\mathrm{,}}{{M}^*}} ) = \frac{{{{P}^*}\ \cdot \ {{M}^*}}}{{{{{| {| {{{P}^*}} |} |}}_2}\ {{{| {| {{{M}^*}} |} |}}_2}}}$. The intuition behind using cosine similarity is that pocket-ligand pairs with stronger binding affinities tend to exhibit higher cosine similarity values and are therefore closer in the embedding space.

#### Data augmentation

To assess the effects of data augmentation, we trained the model under four augmentation settings: no augmentation, simple oversampling, random augmentation, and SHAP-guided augmentation. In the no-augmentation setting, the model was trained on the original dataset. In the oversampling setting, samples from substrate classes with fewer than 10 were repeatedly sampled without changing their sequences or substrates. In the random augmentation setting, additional ABP sequences were generated by BLOSUM-guided residue substitutions at randomly selected positions. In the SHAP-guided augmentation setting, additional ABP sequences were generated using the same BLOSUM-guided substitution strategy, except that substitutions were restricted to three low-SHAP positions with relatively small effects on model output. Each dataset was used to train the same model in a five-fold cross-validation framework. Validation accuracies from the five folds were summarized as mean ± SD. Group distributions were first assessed with the Shapiro-Wilk test, and pairwise differences were subsequently analyzed using two-sided paired *t*-tests.

#### Training

The model was trained to predict the affinity between pockets and ligands, with the loss computation depending on the training dataset. To enhance the cross-modal alignment effect, we adopt a contrastive learning framework that optimizes feature representations by simultaneously attracting genuine binding pairs in the embedding space while repelling nonbinding counterparts. Specifically, we define positive and negative sample pairs based on a batch of samples: given *N* protein pockets $\{ {P_i^*} \}_{i = 1}^N$ and their corresponding *N* molecules $\{ {M_j^*} \}_{j = 1}^N$, we combine them into ${{N}^2}$ pocket-molecule pairs, where *i, j* ∈ [1*, N*]. A pair is considered positive if *i* = *j* or if the label of the pocket$\ {{y}_i}$ matches the label of the molecule$\ {{y}_j}$; it is considered negative if *i*≠*j* and ${{y}_i}\neq{{y}_j}$. Based on this, we use two complementary loss terms as defined in DrugCLIP: Pocket-to-Mol loss and Mol-to-Pocket loss [50]. The former represents the likelihood that a given protein pocket$\ P_i^*$​ exhibits higher similarity to its positive molecules than to all negative molecules:


(11) \begin{eqnarray*}
L_i^{p\to m} = \ - \frac{1}{{\left| {{{P}_i}} \right|}}\mathop \sum \limits_{j\in P(i)} \log \frac{{{\mathrm{exp}}\left( {{{s}_{ij}}/\tau } \right)}}{{\mathop \sum \nolimits_{k = 1}^N {\mathrm{exp}}\left( {{{s}_{ik}}/\tau } \right)}},
\end{eqnarray*}

where$\ {{P}_i} = \{ {j\ {\mathrm{|}}\ {{y}_i} = {{y}_j},\ j\in [ {1,\ N} ]} \}$ denotes the set of indices of all positive molecules for protein pocket $P_i^*$, and${{s}_{ij}} = \hat{p}( {{\mathrm{P}}_i^*{\mathrm{,}}M_j^*} )$ represents the normalized cosine similarity.

The latter defines the likelihood that a given molecule$\ M_j^*$ exhibits higher similarity to its positive pockets than to all negative pockets:


(12) \begin{eqnarray*}
L_j^{m\to p} = \ - \frac{1}{{\left| {{{P}_j}} \right|}}\mathop \sum \limits_{i\in P\left( j \right)} \log \frac{{{\mathrm{exp}}\left( {{{s}_{ij}}/\tau } \right)}}{{\mathop \sum \nolimits_{k = 1}^N {\mathrm{exp}}\left( {{{s}_{kj}}/\tau } \right)}}.
\end{eqnarray*}

In both equations, τ denotes the temperature parameter controlling the softmax distribution. Combining the two losses, we obtain the final loss function for a batch:


(13) \begin{eqnarray*}
L = \frac{1}{{2N}}\left( {\mathop \sum \limits_{i = 1}^N L_i^{p\to m} + \mathop \sum \limits_{j = 1}^N L_j^{m\to p}} \right).
\end{eqnarray*}

This bidirectional objective, calibrated by temperature scaling, directly optimizes the discriminative boundaries between paired instances. Such a formulation makes the loss particularly suitable for supervised contrastive learning in tasks requiring precise interaction modeling. The detailed implementation specifications and parameter configurations are provided in Supplementary Table S1.

#### Probability calibration of model predictions

In the inference stage, DeepAden applied a kernel density estimation (KDE)-based calibration method to convert cosine similarity scores into statistically meaningful confidence probabilities [51]. Using the augmented training data, it computes the similarity between each protein pocket and all molecules to obtain the empirical similarity distributions for positive and negative pairs. These two distributions are modeled separately in a non-parametric manner using Gaussian–kernel KDE, where the bandwidth is set by Scott’s rule and further scaled by a smoothing factor (*α* = 2) to yield a smoother and more robust estimate. During inference, DeepAden calculates the cosine similarity between a query protein pocket and all molecular embeddings in the library, ranks the candidate molecules in descending order of similarity, and then uses the calibrated positive and negative similarity densities within a Bayesian framework to map each similarity score to a posterior probability of being a positive sample. In this way, raw similarity scores are converted into statistically interpretable confidence scores, providing reliable quantification of prediction uncertainty.

### Usage of DeepAden web server

The DeepAden web server accepts input in the form of amino acid sequences in FASTA format, genomic files in FASTA format, and GBK files of biosynthesis gene clusters. Users can adjust the top-k parameter to select the top *k* predictions for each sequence (default *k* = 3). In addition to our predefined database of 223 substrate molecules, users may upload their own custom molecular database as a CSV file with columns labeled “label” and “smiles” (canonical SMILES format is recommended). To enhance the user experience, after model evaluation, we retrained DeepAden using all available data, including 4545 training data and 201 A-domain sequences from the evaluation dataset, resulting in the model weight file “all.weight”. To ensure the reproducibility of the results in this work, we also provide the model weights used during evaluation, stored as “benchmark.weight”.

The prediction results show the top *k* predicted substrates together with their confidence scores, which range from 0 to 1 and are interpreted independently. When the top-ranked prediction has a low score (<0.5), the correct substrate is often still found among the top three candidates, albeit with a relatively low score. In cases with multiple substrates, the model usually assigns the highest confidence to the primary substrate, whereas secondary substrates may also appear among the top three candidates with lower scores. Results can be downloaded in several formats, including CSV and JSON, for downstream analysis. Users may still access previously generated results through the corresponding HTML results pages, provided that the page URLs are retained for up to 15 days. Although DeepAden can identify NRPS BGCs, we recommend using antiSMASH alongside DeepAden for more comprehensive BGC visualization.

### Experimental validation

#### Actinomycete material, culture conditions, and DNA preparation

*Streptomyces hygroscopicus* OsiSh-2 (GenBank accession JBPQZZ000000000) [52] was originally isolated from the sheath of healthy rice (*Oryza sativa* cv. Gumei 4) collected in Liuyang, Hunan Province, China. For the primary cultivation task, the strain was grown on Gauze agar plates at 28°C for 5 days. A seed culture was subsequently prepared by inoculating the strain into liquid Gauze medium, followed by incubation at 28°C with agitation at 180 rpm for 5 days. The cultured broth was then transferred to Gauze plate medium for solid-state fermentation at 28°C for 7 days. High-quality genomic DNA of *S. hygroscopicus* OsiSh-2 was extracted using a standardized protocol referred to Huang *et al*. [24].

#### LC-MS/MS analysis and molecular networking

Crude extracts obtained from different fermentation methods were subjected to UPLC-QTOF-MS/MS analysis. Chromatographic separation was performed on an Agilent 1290 Infinity II UPLC system coupled to an Agilent 6546 QTOF mass spectrometer equipped with a diode array detector (DAD) and operated in positive ion mode. Samples (5 μl injection volume) were separated on a Phenomenex Kinetex C18 column (1.7 μm particle size, 100 × 2.1 mm, 100 Å) at a flow rate of 0.3 ml/min. The mobile phase consisted of (A) 0.1% formic acid in water and (B) 0.1% formic acid in acetonitrile (ACN), using the following gradient program: 20% B (0-2 min), a linear increase to 100% B (2-14 min), held at 100% B (14-16 min), followed by re-equilibration at 20% B for 2.8 min (total run time: 18.8 min). The spray chamber was operated under the following conditions: nebulizer gas flow, 5 L/min; drying gas flow, 5 L/min; drying gas temperature, 200°C; sheath gas temperature, 350°C; and sheath gas flow, 11 L/min. MS and MS/MS data were collected in auto MS/MS (data–dependent) mode over an m/z range of 100-3000 for both MS and MS/MS. Precursor ions exceeding an absolute intensity threshold of 2000 counts were selected for fragmentation, using a precursor mass tolerance of ± 20 ppm and a dynamic exclusion window of 0.2 min. Collision–induced dissociation was carried out with stepped collision energies of 10, 20, and 40 eV.

Raw LC-MS/MS data derived from the OsiSh-2 fermentation extracts were processed using the Global Natural Products Social Molecular Networking (GNPS) platform [53]. For metabolite annotation, feature tables and corresponding MS/MS spectra were exported from GNPS for manual inspection. Accurate-mass MS data (typically <5 ppm error) and isotopic patterns were first used to propose molecular formulas. MS/MS spectra were then compared against GNPS spectral libraries and to reference spectra reported in the literature when available, and putative structures were further evaluated by searching NPAtlas. Molecular networks were constructed and visualized using Cytoscape v3.9.1 [54] with the following parameters: precursor and fragment ion mass tolerances of 0.02 Da, a minimum cosine score of 0.6, at least two matched fragment ions, and a minimum cluster size of 1. Detailed MS/MS-based structural rationale for the octaminomycin and nyuzenamide families is provided in the Supplementary Figs S1 and S2.

## Results

### Overview of DeepAden

**Figure 1.**
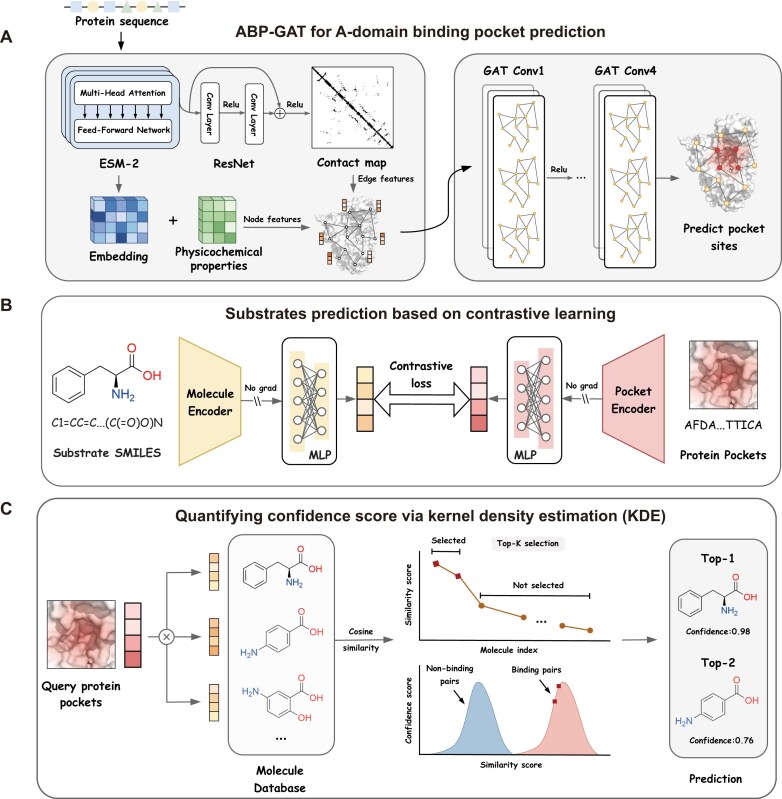
Overview of DeepAden. (**A**) A-domain binding pocket prediction with ABP-GAT. The protein sequence is encoded by ESM2, and residue embeddings are concatenated with physicochemical features to form node representations. A residue-residue contact map (matrices indicating spatial proximity between residue pairs), predicted from ESM2 features by a small ResNet-based module (Supplementary File), defines graph edges. A GAT assigns pocket–forming probabilities to residues and outputs the predicted substrate-binding pocket. Conv Layer: convolutional layer; ReLU: rectified linear unit activation function. (**B**) Substrate prediction by contrastive learning. A pocket encoder (ABP-ESM2) and a molecule encoder (MoLFormer) produce embeddings that are further projected by trainable MLP heads. The model pulls embeddings of true pocket-substrate binding pairs together and pushes apart non-binding pairs in a shared embedding space. No grad: no gradient. (**C**) Confidence estimation using KDE. Separate KDEs are fitted to the cosine similarities of true binding pairs (positive distribution) and non-binding pairs (negative distribution) from training data. The similarity of a new pocket-substrate pair is evaluated under this KDE: scores in high–density regions are assigned higher confidence, whereas low-density scores are considered less reliable.

To identify pocket residues involved in substrate recognition, we first developed ABP-GAT, a module that integrates ESM2 with a GAT. ABP-GAT was designed to locate potential substrate-binding pockets using only the amino acid sequence, without relying on explicit structural data. It first transformed each sequence into a protein graph, where individual residues served as nodes. Each node combined two types of information, namely the sequence embeddings learned by ESM2 and the physicochemical properties of each residue. To determine how residues were connected within this graph, we employed a residual neural network (ResNet) that took the attention map from ESM2 as input to predict residue-residue contacts. The predicted contact map defined the edges between residues, thereby establishing the overall graph structure. Once the graph was constructed, ABP-GAT was trained to identify residues forming the substrate-binding pocket (Fig. 1A).

After identifying the binding-pocket residues, DeepAden predicted substrate specificity by matching each pocket with potential substrates. This task was formulated as a retrieval problem, where the model learned to pair each pocket with its true substrate among many candidates. To achieve this, we used two pretrained encoders: A fine-tuned ESM2 for ABP feature representation (ABP-ESM2) for pocket sequences and MoLFormer for substrate SMILES strings (see the “Materials and methods” section). Their outputs were passed through trainable MLP projection heads, which were optimized so that embeddings of interacting pocket-substrate pairs were pulled closer together, whereas embeddings of non-interacting pairs were pushed farther apart in the shared embedding space (Fig. 1B). To complement this model, we trained an independent pairwise random forest classifier on pocket-substrate features and used SHAP to identify key residues in the A-domain pocket. Residues with high SHAP importance were kept fixed, whereas the three positions with the lowest SHAP importance in each pocket were selectively mutated using BLOSUM-62 substitutions. This SHAP-guided augmentation generated new, biologically plausible pocket-substrate pairs and effectively expanded the training space for DeepAden.

Finally, we quantified prediction confidence by modeling the distribution of cosine similarity scores between pocket and substrate embeddings using KDE [51]. This non-parametric estimate allowed us to distinguish score ranges corresponding to densely populated, reliable matches from sparse, ambiguous ones and used these density-based thresholds as a confidence criterion to distinguish high-confidence substrate matches from uncertain cases (Fig. 1C). More detailed workflow could refer to Supplementary Fig. S3. To train and evaluate this framework, we curated datasets from two recent studies, NRPStransformer (4430 entries) and PARAS (3257 entries), and further expanded them by manual literature search, yielding a final training set of 4545 A-domain sequences from the bacterial phylum, fungal kingdom, metazoan kingdom, and plant kingdom (Supplementary Fig. S4).

### DeepAden achieves stable binding pocket prediction across evolutionary divergence via GAT

**Figure 2.**
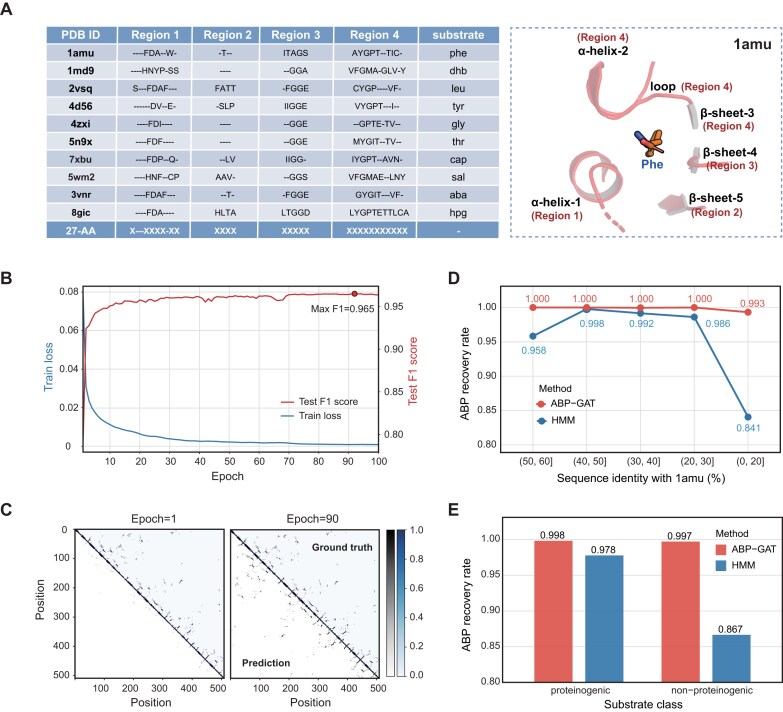
Performance of ABP-GAT model for binding pocket prediction. (**A**) Ten A-domain cocrystal structures with bound substrates are used to define a canonical 27-residue A-domain binding pocket (27-AA ABP). Pocket residues are defined as those within 6 Å of the substrate and are distributed across four conserved secondary structure regions. (**B**) Residue contact map training curve. A ResNet-based contact-map predictor trained on 78 A-domain crystal structures reached a peak F1-score of 0.965 on the test set, showing accurate learning of residue-residue contacts. (**C**) Residue contact maps at early and late training stages. Predicted contact maps (lower triangles) at early and late training stages progressively approach the experimental contact maps (upper triangles), illustrating that the predictor captures the underlying structural constraints. (**D**) ABP recovery rate comparison across identity ranges. For 4545 A-domain sequences, ABP-GAT (red) and a 1AMU-based HMM method (blue) are evaluated for recovery of the complete 27-AA ABP. Sequences are binned by their identity to the reference structure 1AMU, and ABP-GAT maintains high recovery across all identity ranges. (**E**) ABP recovery rate comparison by substrate class. ABP-GAT and the HMM method are further compared on A-domains recognizing proteinogenic and nonproteinogenic substrates. ABP-GAT maintains high ABP recovery in both classes, while the HMM performs substantially worse on nonproteinogenic substrates.

The traditional coding approaches such as 10-AA [7] and its derived 13-AA [55] and 17-AA [56] SCCs face limitations in terms of predicting diverse substrates due to evolutionary divergence in A-domain structures. The 34-AA ABP represented a major advancement by incorporating structural elements within an 8 Å substrate radius in GrsA-PheA (PDB: 1AMU) [8]. However, SCC and ABP definitions derived from this single GrsA–PheA cocrystal structure show limited generalizability across phylogenetically diverse A-domains and nonproteinogenic substrates. To obtain a more broadly applicable and structurally grounded ABP definition, we carried out a comprehensive structural analysis of 10 available A-domain-substrate cocrystal structures that contain bound amino-acid-like ligands without AMP, including both proteinogenic and nonproteinogenic substrates (Fig. 2A). Noncovalent interactions that mediate substrate recognition, such as hydrogen bonds, salt bridges, and van der Waals contacts, typically operate within ∼6 Å [57, 58, 59], and several recent protein-ligand modeling frameworks (e.g. DrugCLIP [50]) have adopted a 6 Å ligand-centered radius to define pocket regions. Guided by this biophysical rationale, we systematically compared pocket definitions based on residues within either a 6 Å or a traditional 8 Å radius of the bound substrate across these 10 cocrystal complexes. By mapping all 10 structures to a common sequence-structure alignment, we defined two ABP sets as the union across all structures of contacting residues under each distance cutoff: 27-AA ABP comprising all positions that lie within 6 Å of the substrate (Fig. 2A), and 49-AA ABP comprising all positions that lie within 8 Å (Supplementary Table S2). Because the 8 Å cutoff captures a broader shell of substrate-proximal residues than the 6 Å cutoff, the resulting 49-AA ABP includes more positions than the 27-AA ABP. These two sets provide directly comparable pocket definitions derived from the same structural corpus but using different distance thresholds.

To overcome the limitations of HMM algorithms, which rely exclusively on primary sequence alignments for binding-pocket prediction, we next exploited both sequence and structural information from 78 published A-domain crystal structures to train a ResNet-based residue contact map predictor (Fig. 2B). This model achieved an F1 score of 0.965 on the test dataset and accurately captured residue-level interaction patterns within A-domains, as illustrated by the convergence of predicted and experimental contact maps over training (Fig. 2C), providing residue-level structural constraints for downstream ABP prediction. Using these predicted contact maps as edge information and protein language model embeddings as node features, we developed ABP-GAT models under both the 6 Å and 8 Å pocket definitions to assess how the distance cutoff affects residue-level predictability. Under leave-one-out cross-validation, the 6 Å and 8 Å-based ABP-GAT models achieved mean residue-level F1 scores of 0.978 ± 0.033 and 0.952 ± 0.068, respectively, with 95% bootstrap confidence intervals of 0.970-0.985 and 0.937-0.966. We then further evaluated the two pocket definitions on a curated set of 4545 A-domains using the ABP-set recovery rate, defined as whether the complete ABP residue set could be assigned to an A-domain rather than whether each predicted residue was correct individually. Under the 27-AA definition, the ABP-set recovery rate of ABP-GAT was 0.998, whereas under the 49-AA definition it decreased to 0.939, indicating that the 27-AA definition is more consistently recoverable and therefore better suited for subsequent modeling. Notably, all 27-AA are included within 34-AA defined by Rausch *et al*. [8] and extend the canonical 10-AA defined by Stachelhaus *et al*. [7] (Supplementary Table S3).

We next benchmarked ABP-GAT against an HMM-based method under the 6 Å-based 27-AA ABP definition. To this end, we evaluated the curated set of 4545 A-domains that share 0-60% sequence identity with the reference A-domain GrsA–PheA (1AMU) and cover diverse taxonomic origins. For the HMM baseline, we used the NRPSPredictor2 profile built on 1AMU to infer binding-pocket residues. For both methods, performance was assessed using the same ABP-set recovery criterion described earlier. Across all sequence-identity bins within the 0-60% range, the ABP-set recovery rate of the HMM approach declined sharply as identity to 1AMU decreased, whereas ABP-GAT maintained consistently high recovery rates in every bin (Fig. 2D). We further stratified A-domains by substrate class and observed that ABP-GAT achieved similarly high recovery rates for both proteinogenic and nonproteinogenic substrates, while the HMM model performed substantially worse for nonproteinogenic substrates (Fig. 2E). These results indicate that the learned graph-based model generalizes better than the 1AMU-based HMM profile, particularly for evolutionarily distant A-domains and those specific for nonproteinogenic substrates.

### Conservative and functional residue recognition through evolutionary and SHAP analyses

**Figure 3.**
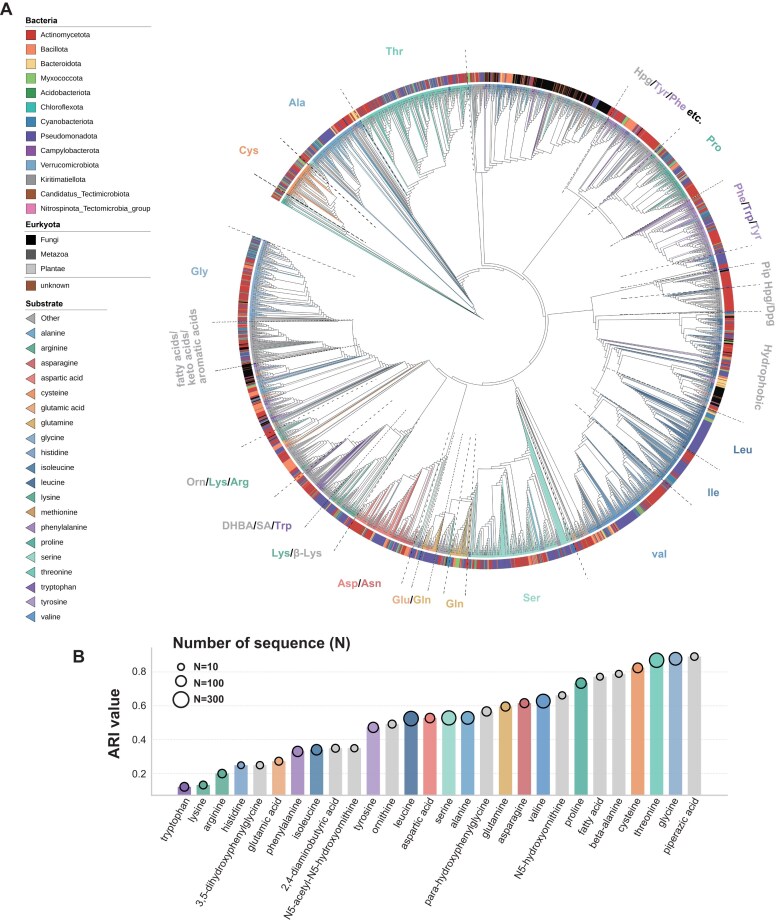
Evolutionary relationships and substrate specificity of ABP sequences. (**A**) Circular phylogenetic tree constructed from a curated dataset of 4545 ABP sequences. Each leaf represents a single A domain, with branches colored by substrate and the outer ring indicating taxonomic affiliation. (**B**) Bar plot showing the ARI, quantifying how well clusters of bacterial ABP binding–pocket sequences in the phylogenetic tree agree with their substrate labels. The tree was cut at a fixed height (H = 2.0 substitutions) to define clusters, which were compared with substrate labels using ARI (1 = perfect agreement, ≈0 = random); point size indicates the number of sequences collected for each substrate label.

Employing the ABP-GAT model with sequence-based inputs, we obtained 4545 27-AA ABP sequences with known substrate annotations. To assess how strongly these short ABP segments encode substrate information, we constructed a phylogenetic tree based solely on the 4545 27-AA ABP sequences alignment (Fig. 3A) and examined how this “pocket-level” phylogeny relates to organismal classification. Visual inspection of the tree revealed substrate-enriched sectors around the perimeter, for example, regions dominated by glycine, threonine, cysteine, alanine, serine, branched-chain hydrophobic residues (Val/Ile/Leu), and basic residues (Lys/Orn/Arg). Within these substrate-enriched regions, however, sequences from multiple bacterial phyla and from eukaryotes are intermingled, and long branches that are simultaneously dominated by a single phylum and a single substrate label are relatively uncommon. This pattern suggests that, at the level of the substrate-binding pocket, sequence similarity is shaped primarily by substrate-related evolutionary pressures and is only weakly aligned with broad organismal lineages. In particular, fungal ABPs (black ring) are scattered across multiple substrate-enriched sectors and frequently co-occur with bacterial ABPs that share the same substrate label (Fig. 3A). We then quantified how tightly each substrate label is clustered on the 27-AA ABP tree using an adjusted Rand index (ARI) [48] analysis (Fig. 3B). Substrates such as piperazic acid and the small or polar amino acids glycine, threonine, and cysteine exhibited high ARI values, indicating that their corresponding ABPs are concentrated within relatively compact regions of the tree. In contrast, substrates with lower ARI values are more widely dispersed across the phylogeny, consistent with more heterogeneous or promiscuous binding pockets.

**Figure 4.**
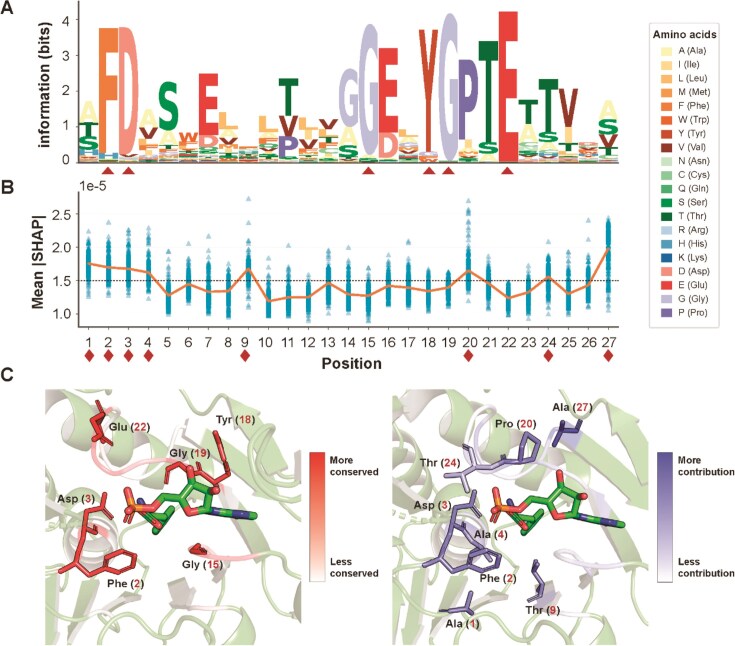
Conservation and substrate-specificity determinants in ABP. (**A**) Sequence logo and conservation profile of 4545 ABP sequences across the 27-AA binding-pocket positions. Triangles mark higher conserved residues. (**B**) Mean absolute SHAP value (|SHAP|) for each of the 27 ABP positions in pairwise random-forest classifiers distinguishing substrate classes, representing the contribution of each position to substrate specificity. Each point corresponds to the mean |SHAP| value for a single substrate class at the indicated position. Diamonds indicate positions with the higher contributions. (**C**) Conservation scores (left) and SHAP-based contribution scores (right) mapped onto the crystal structure of GrsA-PheA (PDB: 1AMU), highlighting residues that are both highly conserved and strongly associated with substrate specificity.

We observed that positions 2, 3, 15, 18, 19, and 22 are more highly conserved among labeled pockets (Fig. 4A), indicating a limited set of tolerated amino acids at these sites. Such conservation suggests that these positions contribute to maintaining the pocket architecture and/or to the recognition of particular substrate types. Because substrate annotations in our dataset are strongly imbalanced, further analyses are needed to clarify the substrate-specific contributions of individual positions. To move from these global patterns to a residue-resolved view of substrate recognition, we next quantified the contribution of each ABP position using model-agnostic feature attribution. Specifically, we leveraged SHAP to interpret predictions from machine-learning models trained on ABP sequences. Building on this approach, we fine-tuned the ESM2-650M model on a curated dataset of 191 303 ABP sequences, yielding a model referred to as ABP-ESM2. This model generated context–aware positional embeddings that exhibited clearer class separation in embedding space than the original ESM2 model, while effectively preserving positional information critical for downstream analyses. For the labeled subset of 4545 ABP sequences, we applied an oversampling strategy combined with 5-fold cross-validation to train random-forest classifiers on pairwise substrate-versus-non-substrate discrimination tasks, fitting a separate classifier for each substrate class. SHAP values were then computed for each substrate-specific model directly on the ABP-ESM2 embeddings. Across all substrate classes, the SHAP analysis highlighted eight positions (1, 2, 3, 4, 9, 20, 24, and 27) with the largest overall contributions (Fig. 4B and C). These positions cluster around non-carboxyl substrate moieties, side-chain interaction zones, and the only loop segment lining the pocket, indicating that contacts in these regions are a major source of discriminative information for substrate specificity. In particular, the loop-embedded positions 20 and 24 are located near the pocket entrance, suggesting that this flexible segment helps shape the local binding environment and modulate how different substrates are accommodated. The highly conserved positions 15, 19, and 22 show comparatively low SHAP contributions, suggesting that they provide little discriminative information for distinguishing substrates in our models and are more likely involved in maintaining the overall pocket scaffold than in fine-tuning substrate preference. Although positions 2 and 3 are highly conserved, they still help distinguish ABPs specific for proteinogenic substrates from those recognizing nonproteinogenic substrates (Supplementary Fig. S5). Together, these patterns indicate that combining conservation with SHAP-based feature attribution distinguishes structurally important pocket residues from those that directly encode substrate type.

**Figure 5.**
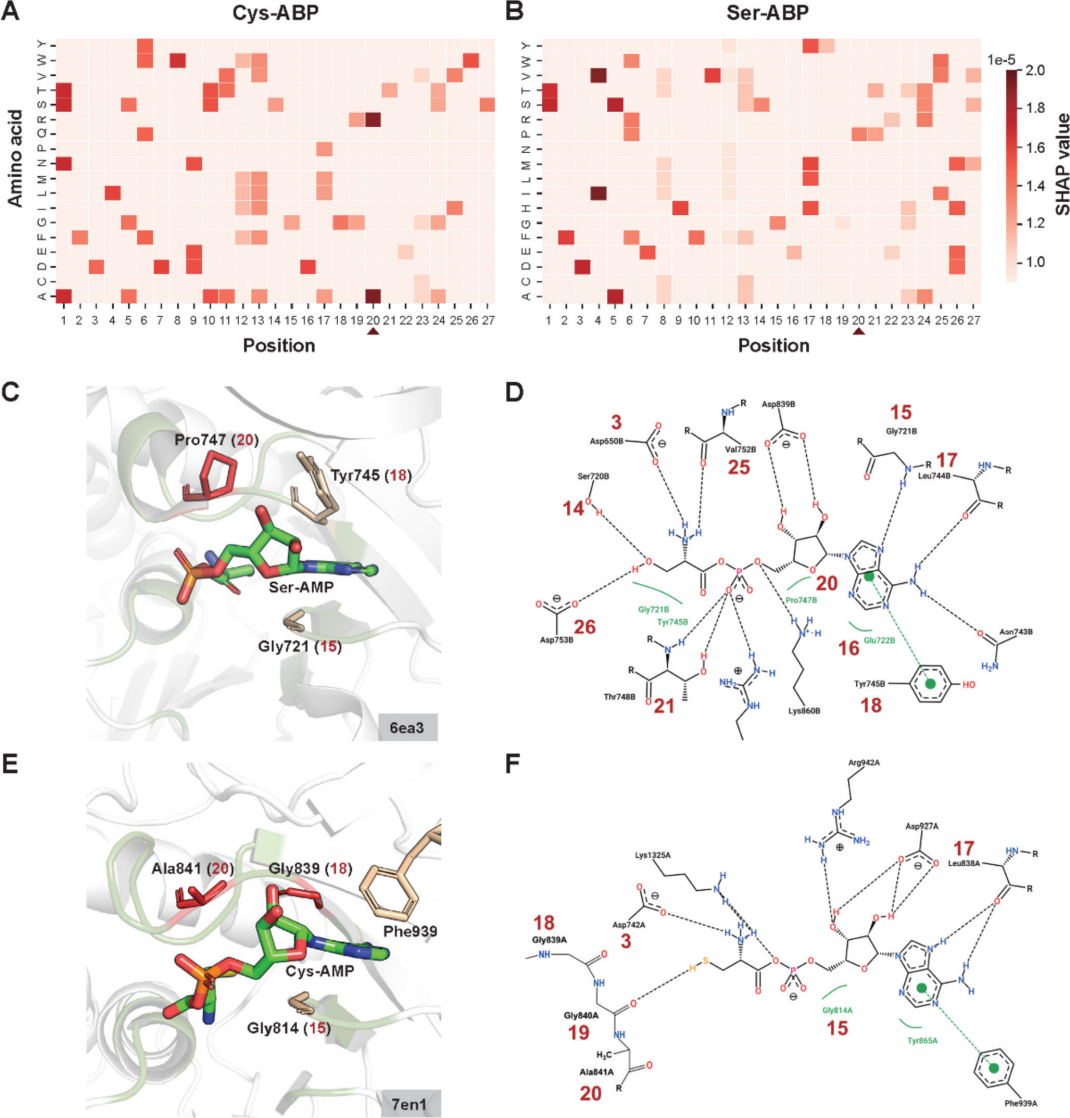
Structural and interaction analysis of functional residues in ABP based on PchE-Cys and FscH-Ser crystal structures. (**A, B**) Heatmaps of positive SHAP values highlighting amino-acid contributions at each binding-pocket position for Cys-ABPs and Ser-ABPs, respectively, for the sequence clusters shown in Fig. 3A. (**C**) Structural representation of contributing residues within the ABP of FscH-Ser (PDB: 6ea3). The bound ligand is Ser-AMP, containing an adenine ring and a pentose sugar. Red numbers in parentheses indicate positions in the 27-AA ABP, and black labels indicate amino acids and their positions in the protein sequence. (**D**) Two-dimensional interaction diagram of FscH-Ser (PDB: 6ea3). Interactions between protein and ligand are shown as dashed lines, with both interacting residues and the ligand drawn as structural formulas. Hydrophobic contacts are indicated by highlighted hydrophobic regions of the ligand and the labels of contacting residues, and dashed lines between aromatic rings denote π-π stacking interactions. (**E**) Structural representation of contributing residues within the ABP of PchE-Cys (PDB: 7en1). The bound ligand is Cys-AMP; residue annotations follow the same convention as in panel (C). (**F**) Two-dimensional interaction diagram of PchE-Cys (PDB: 7en1), with interaction types annotated as in panel (D).

In DeepAden, SHAP–guided augmentation both guides position-specific substitutions for data augmentation and provides a framework for systematically identifying substrate-specific residues across characterized A-domains. In the 27-AA ABP phylogeny (Fig. 3A), Cys-ABPs and Ser-ABPs form distinct clades, even though L-Cys and L-Ser differ only subtly in side-chain size and polarity. This observation prompted us to ask whether SHAP could resolve the fine–scale ABP differences that underlie this separation. For the clarity of the following description, the superscript numbers preceding the amino acid residues indicate their positions within the ABP sequence alignment results, whereas the numbers following the residues correspond to their positions in the resolved PDB structure. Class-specific SHAP heatmaps for Cys-ABPs and Ser-ABPs revealed pronounced differences, with position 20 showing the largest substrate-dependent shift in SHAP values and corresponding to ^20^Ala in Cys-ABPs and ^20^Pro in Ser-ABPs (Fig. 5A). High-resolution structural comparisons between the Cys-AMP-bound (PDB: 7en1) and Ser-AMP-bound (PDB: 6ea3) complexes revealed distinct ligand coordination mechanisms consistent with these SHAP-highlighted differences (Fig. 5C and F). In Ser-ABPs, ^20^Pro rigidifies the loop at the pocket entrance and packs against the ribose-phosphate moiety, restricting its conformational flexibility (Fig. 5C and D). In Cys-ABPs, the corresponding ^20^Ala, together with neighboring ^18^Gly, creates a more flexible local environment that permits alternative orientations of the ribose-phosphate group (Fig. 5E and F). This difference in loop flexibility is accompanied by a rearrangement of adenine interactions. In Cys-ABPs, the adenine ring adopts an "edge-to-face" π-π stacking interaction with Phe939 located outside the canonical binding pocket, tilting the ring upward and enabling two hydrogen bonds to form with ^17^Leu (Fig. 5E and F). As a consequence, the ribose moiety is reoriented, with its O4′ atom positioned closer to the adenine ring than in the Ser-ABP complex. The increased local flexibility around positions 18 and 20 in Cys-ABPs also facilitates productive positioning of the Cys thiol group to hydrogen bond with ¹⁹Gly. Together, these case studies suggest that SHAP-identified positions, particularly position 20, correspond to structurally meaningful interaction hotspots that differentiate Cys-ABPs and Ser-ABPs, thereby supporting both the biological plausibility of our attribution analysis and the rationale for constraining data augmentation to positions with low SHAP importance.

### DeepAden achieves SOTA performance in substrate specificity prediction, including nonproteinogenic substrates

Current methods for predicting NRPS A-domain substrates perform poorly on recently identified nonproteinogenic substrates, largely because suitable training sequences are scarce and sequence alignment-based SCC extraction has clear limitations. To increase the effective data for these substrates, we augmented their ABP sequences. Simple oversampling or random perturbations risk overfitting and generate sequences with low biological plausibility, so we instead adopted a SHAP-guided augmentation strategy. Based on SHAP and conservation analyses (Figs 4 and 5), we distinguished residues that were evolutionarily conserved, functionally important, or located in interaction hotspots from residues that were weakly conserved, have minimal impact on model predictions, and lie outside structurally validated binding regions. Only the latter positions were selected for *in silico* mutagenesis, as substitutions at these sites are more likely to be tolerated without altering substrate specificity. Guided by observed substitution patterns and physicochemical similarity, we introduced small, conservative mutations at these low-influence positions, thereby expanding the local sequence space around under-represented nonproteinogenic substrates while preserving key recognition features (Supplementary Fig. S3). The augmented dataset was then used in the subsequent contrastive learning stage to enhance the model’s discriminative ability.

**Figure 6.**
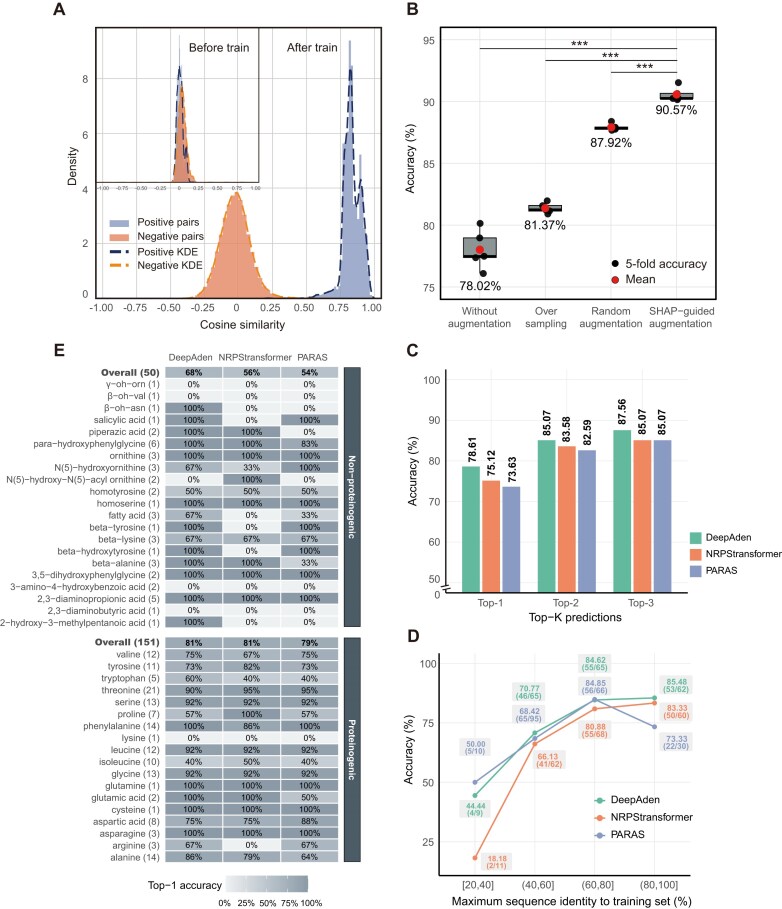
Model performance evaluation of DeepAden. (**A**) Positive and negative pair distribution of training set. Cosine similarity distributions of positive (true pocket-substrate) and negative pairs before and after contrastive learning. After training, positive and negative pairs form two well-separated modes. (**B**) Five-fold cross-validation accuracy on the validation set for four augmentation strategies. SHAP-guided augmentation achieved the highest accuracy (90.57% ± 0.57%), compared with random augmentation (87.92% ± 0.37%), oversampling (81.38% ± 0.79%), and no augmentation (78.02% ± 1.58%). (**C**) Top-3 accuracy on the benchmark dataset. On an independent benchmark dataset of 201 A-domains, DeepAden achieved slightly higher Top-1, Top-2, and Top-3 accuracies than NRPStransformer and PARAS. (**D**) Model performance across different homology levels. Benchmark datasets were grouped according to their maximum sequence identity to the training set of each model. Accuracy decreases as sequence identity drops, but DeepAden maintains competitive performance across different homology levels. (**E**) Model performance on different substrate types. For proteinogenic substrates (*n* = 151), all three models performed similarly. For nonproteinogenic substrates (*n* = 50), DeepAden showed higher accuracy than NRPStransformer and PARAS, suggesting an advantage in this category, although the limited sample size warrants cautious interpretation.

In the second stage, we trained the substrate prediction model within a contrastive learning framework using the augmented dataset. The data were split into training and validation sets at an 8:2 ratio, and five-fold cross-validation was used for model training and hyperparameter tuning. After identifying the optimal hyperparameters, we retrained the model on the full dataset to obtain the final weights. The trained DeepAden model captured the characteristic interaction patterns between the A-domain binding pockets and substrate molecules, effectively distinguishing positive sample pairs (pocket-substrate pairs) from negative sample pairs (pocket-non-substrate pairs) (Fig. 6A). In the embedding space, contrastive learning successfully shortened the distance between substrate molecules and their corresponding pockets (Supplementary Fig. S6). We next compared four augmentation strategies, namely no augmentation, simple oversampling, random augmentation, and SHAP-guided augmentation. Across five-fold cross-validation, SHAP-guided augmentation improved validation accuracy by 2.65% (*p* = 9.16 × 10^-5^), 9.19% (*p* = 1.16 × 10^-5^), and 12.55% (*p* = 9.81 × 10^-5^). These results indicate that SHAP-guided augmentation provides a clear performance advantage over unguided or naive data augmentation strategies.

We next benchmarked DeepAden against two recently developed A-domain substrate prediction tools, NRPStransformer [35] and PARAS [15]. The benchmark set contained 201 A-domain entries with no overlap with the training data of the assessed tools. Of these, 155 sequences were taken from the NRPStransformer benchmark, and 46 sequences were manually curated from literature corresponding to newly added MIBiG 4.0 entries [33] (Dataset S3, Supplementary Fig. S7). On the 201-sequence benchmark, DeepAden achieved slightly higher top 3 accuracy than NRPStransformer and PARAS by 2.5-3.3% in the overall comparison (Fig. 6C). Pairwise bootstrap analysis yielded positive overall top 3 accuracy differences for DeepAden relative to both NRPStransformer and PARAS, with corresponding confidence intervals that did not cross zero. Nevertheless, all three tools remained close in performance (Supplementary Table S4).

To assess the generalization capacity of each tool, particularly when handling A-domain sequences with low homology to their respective training sets, we calculated, for each of the 201 test sequences, its highest sequence identity to the training sequences of each tool and used this value as its similarity to that tool’s training data. Prediction accuracy was then evaluated across sequence identity intervals. Across all three tools, accuracy declined with decreasing sequence identity to the corresponding training data, indicating reduced predictive performance in more remote sequence space. In the high-similarity range (80, 100], DeepAden achieved the highest accuracy of 85.48% (53/62), with NRPStransformer performing comparably well. In the moderate–similarity range (40, 80], the performance of all three tools was comparable, though accuracy decreased relative to the high-similarity interval. In the low-similarity range, predictive performance declined sharply for all models. Here, PARAS achieved 50.0% accuracy (5/10), followed by DeepAden at 44.44% (4/9), whereas NRPStransformer reached only 18.18% (2/11) (Fig. 6D). However, the small number of test sequences in this interval limits the statistical power of these comparisons. Pairwise bootstrap analysis of the identity-stratified results showed that DeepAden had a more favorable overall performance profile than NRPStransformer across sequence identity intervals, whereas differences between DeepAden and PARAS were not clearly supported (Supplementary Table S4).

Finally, we evaluated the ability of the three tools to predict two broad substrate categories: proteinogenic and nonproteinogenic amino acids (Fig. 6E). For proteinogenic substrates, the methods showed broadly similar performance, and the small numerical differences observed for DeepAden relative to NRPStransformer and PARAS were not supported by bootstrap analysis, as all corresponding 95% confidence intervals included zero. For nonproteinogenic substrates, DeepAden showed higher accuracy than both comparison methods, and pairwise bootstrap analysis supported a positive advantage over NRPStransformer and PARAS. However, because the nonproteinogenic subset was limited in size, the confidence intervals were wide, and this result should therefore be interpreted as a supported advantage, rather than definitive superiority.

### DeepAden supports substrate-based assignment of orphan NRPS BGCs

As a proof-of-concept application of DeepAden-assisted genome mining, we sought to link NRPS BGCs in the rice endophytic actinomycete *Streptomyces hygroscopicus* OsiSh-2 (GenBank accession JBPQZZ000000000) to their corresponding metabolites [52]. We first performed untargeted LC-MS/MS–based metabolomic analysis of culture extracts of *S. hygroscopicus* OsiSh-2 and constructed a GNPS MS/MS molecular network. This analysis revealed two prominent molecular families: one cluster with precursor ions in the m/z 1300-1450 range, and a second cluster with ions between m/z 900 and 1100, each displaying highly similar fragmentation patterns within the respective family (Supplementary Fig. S8). By matching accurate precursor *m/z* values, inferred molecular formulas, and diagnostic MS/MS fragments to literature data, we putatively annotated two NRP families as nyuzenamide (B and D) and octaminomycin (A-D). In the octaminomycin family, the MS/MS spectra showed rich fragment ion coverage, which enabled a detailed interpretation of the peptide backbone directly from the MS/MS data and subsequent comparison with A-domain substrate predictions (Supplementary Fig. S1). In contrast, the nyuzenamide family exhibited limited backbone fragmentation, consistent with previous reports on bicyclic peptides [60, 61], so our annotation primarily relied on accurate mass, molecular formula, and high spectral similarity to published nyuzenamide reference spectra [61, 62], together with the similarity between the candidate BGC and previously reported nyuzenamide clusters (Supplementary Fig. S2).

The BGCs of *S. hygroscopicus* OsiSh-2 were analyzed using antiSMASH v7.0, which identified 12 NRPS/NRPS-like BGCs (Supplementary Table S5). To establish gene cluster-compound correlations, we adopted an A-domain-based strategy that proceeds from metabolite to genome and back to biosynthetic logic. First, we deduced the amino acid building blocks of the two target families from their putative structures inferred from the MS/MS data: nyuzenamides with the core peptide Phe-Pro-Leu-Gly-Trp-Asn-Thr-Hpg-Val-Gly, and octaminomycins with the core peptide Thr-Phe-Leu-Val-Pro-Leu-Tyr(Phe)-Pro(Pip). Notably, given the potential involvement of various modifying enzymes (e.g. isomerases and methyltransferases) during biosynthesis, we focused exclusively on the core scaffold derived from the initially recognized A-domain substrates, without accounting for late-stage tailoring modifications that may generate derivative structures. DeepAden was then used to predict substrate specificities for all A-domains encoded in the candidate NRPS BGCs. For each A-domain, DeepAden independently computed a score for every candidate substrate, allowing multiple plausible substrates to be considered for a single domain when appropriate. By systematically aligning the DeepAden substrate profiles with the amino acid compositions of the target core peptides, we were able to assign one NRPS BGC as the putative nyuzenamide cluster and another as the putative octaminomycin cluster. The GBK files of these two BGCs have been deposited in our GitHub repository.

**Figure 7.**
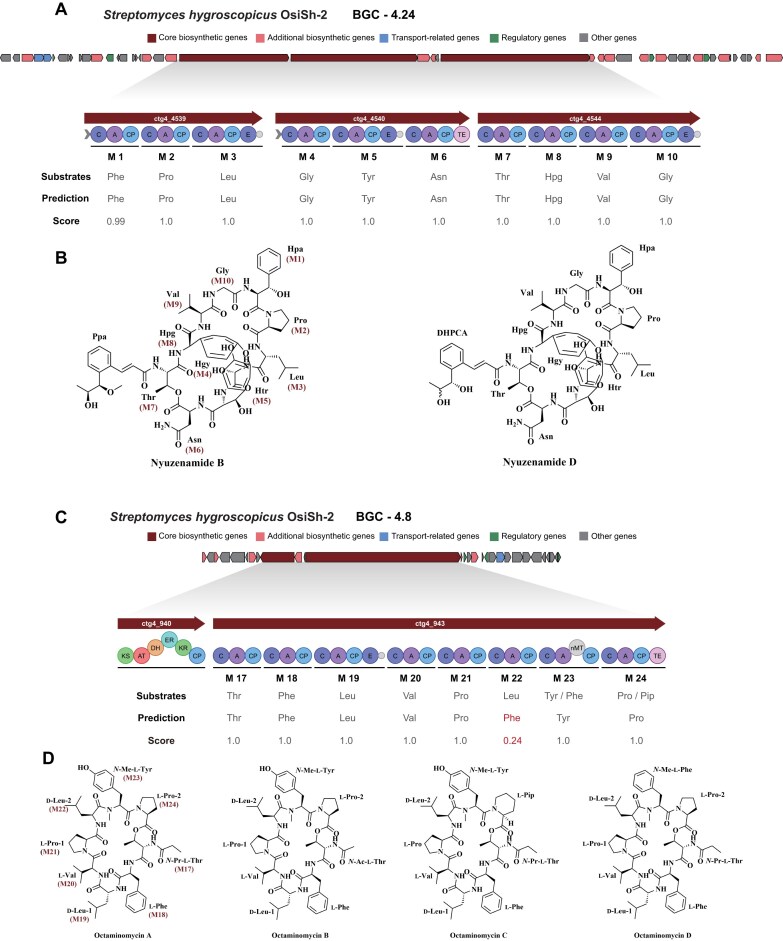
DeepAden-guided, structure-based assignment of orphan NRPS BGCs in *Streptomyces hygroscopicus* OsiSh-2. (**A**) Genomic context and NRPS organization of BGC-4.24 with DeepAden substrate predictions and scores for each A-domain (M1-M9). (**B**) Nyuzenamide B and D structures with their core peptide (Phe-Pro-Leu-Gly-Trp-Asn-Thr-Hpg-Val-Gly) mapped to the corresponding A-domains in BGC-4.24, supporting its assignment as the nyuzenamide BGC. (**C**) Genomic context and NRPS organization of BGC-4.8 with DeepAden substrate predictions and scores for A-domains M17-M24, including positions with experimentally observed alternative substrates (M23: Tyr/Phe; M24: Pro/Pip). (**D**) Octaminomycin A-D with the core peptide [Thr-Phe-Leu-Val-Pro-Leu-Tyr(Phe)-Pro(Pip)] mapped to BGC-4.8. DeepAden recovers the primary substrates and ranks the alternative substrates among the top predicted candidates.

For the nyuzenamide BGC, DeepAden recovered the expected substrate preference at every position of the core peptide scaffold (Fig. 7A and B), thereby supporting a consistent cluster-product assignment at the level of the peptide backbone. In the case of the octaminomycin BGC, the A-domain in module M22 was predicted to incorporate Phe, but with a low confidence score (0.24), whereas the true substrate Leu ranked second with a score of 0.15. Notably, the A-domains in modules M23 and M24 showed recognition of multiple substrates (M23: Tyr/Phe; M24: Pro/Pip). In both modules, the primary substrates (Tyr and Pro) ranked first with confidence scores of 1.0, while the alternative substrates were still captured among the top-ranked predictions but with lower scores (Phe ranked second with 0.18 in M23, and Pip ranked third with 0.02 in M24) (Fig. 7C and D). This pattern indicates that the presence of these alternative amino acids is recognized by the model, albeit with markedly lower confidence than the dominant assignments.

## Discussion

Substrate adenylation by A-domains is essentially a protein-ligand interaction process, requiring precise localization of the binding pocket and accurate substrate pairing for reliable prediction. With the development of deep learning, an increasing number of algorithmic frameworks have been proposed to tackle such interaction prediction tasks, particularly those involving high-dimensional and structured biological data [63]. Recent advances in pretrained PLMs have enabled the integration of structural context into A-domain representation learning. Meanwhile, contrastive learning has proven effective in protein-ligand interaction prediction [26]. In this study, we propose DeepAden, a two-stage framework that first trains a GAT to identify the binding pocket surrounding the target substrate by fusing sequence features with structural embeddings derived from a protein language model. In the second stage, it employs cross-modal contrastive learning to jointly encode pocket-substrate interactions using both protein and chemical language models, effectively addressing challenges arising from evolutionary divergence in A-domain substrate prediction. By integrating these modules, DeepAden provides a practical framework for inferring substrate preferences from A-domain sequences alone, without requiring 3D structural data. This eliminates the dependence on 3D data and paves the way for robust learning across large-scale sequence databases. To support the natural product research community, we developed a user-friendly web server for ABP residue identification and substrate prediction.

Recent methods have started to extract A-domain signatures by aligning structures predicted by ColabFold [15]. However, the large-scale binding–pocket prediction required in our study remains computationally impractical, as generating a single A-domain structure with the AlphaFold3 web server takes ~5 min and the server currently limits users to ~30 submissions per day. At the same time, sequence-alignment-based ABP extraction degrades on evolutionarily divergent A-domains [3], limiting its applicability to nonredundant NRPS repertoires. To address these issues, we developed ABP-GAT, which mitigates the impact of evolutionary divergence by inferring ABPs directly from local pocket environments and maintains robust performance on nonhomologous A-domains with only 30-40% sequence identity to 1AMU. Our analyses further show that a compact 27-AA pocket defined by a 6 Å cutoff is sufficient to support accurate substrate prediction. This 27-AA pocket achieves substrate prediction performance comparable to two state-of-the-art tools, NRPStransformer [35] (which uses the flavodoxin-like subdomain) and PARAS (which uses a 34-AA pocket) [15], indicating that a 27-AA pocket captures enough local information for reliable substrate inference. Moreover, when performance was stratified by global sequence identity, the pocket-based models (DeepAden and PARAS) appeared less sensitive to global homology than the full-length-based NRPStransformer, consistent with their underlying design principles, although a more stringent low-homology benchmark will be required to fully assess this advantage. The ABP extraction method we developed suggests broad applicability and may provide a useful tool for further research. Building on this approach, future studies can investigate the functional mechanisms of these residues across diverse A-domains, which may help improve the accuracy and applicability of substrate-specificity prediction algorithms. To support broader usage, we offer predicted ABPs through both our command-line tools and web server, allowing users to explore the diversity and evolutionary dynamics of pocket sequences. It is worth noting that while our 27-AA pocket within a 6 Å universal boundary balances computational efficiency and generalization, its static boundaries may be limited in their ability to recognize large substrates and accommodate protein conformational changes [64, 65]. Future research should explore dynamic pocket delineation strategies that incorporate molecular docking energy landscapes or residue-substrate contact frequency analyses. Such adaptive methods could enhance the accuracy of binding-interface characterization and improve substrate-specificity prediction performance.

DeepAden employs a contrastive learning-based independent modeling strategy that generates separate predictions and distinct confidence scores for each pocket-substrate pair. This mechanism reduces direct competition among substrates and is, in principle, more compatible with the multi-substrate nature of biochemical reactions at the A-domain level. In contrast, many existing tools utilize softmax functions to normalize probabilities across substrate libraries [12, 15]. Consequently, their mutual-exclusivity assumption can lead to averaged low-probability predictions or a single dominant substrate, which may underrepresent potential multi-substrate usage. However, recent study has illustrated that A-domain-level information alone does not fully determine whether a given amino acid can be incorporated into the final product, as additional constraints arise from the PCP domain and the broader module-level context [66]. This distinction is exemplified by our analyses of the nyuzenamide and octaminomycin BGCs (Fig. 7), where DeepAden recovers most expected substrate preferences and places experimentally observed alternative substrates within the top-3 predictions but assigns them lower confidence than the dominant amino acids. Notably, DeepAden did not achieve perfect agreement for all modules; for example, in octaminomycin module M22, Phe was ranked first, whereas Leu, the substrate supported by the peptide structure, was ranked second. Moreover, because no direct biochemical assays were performed for individual A-domains in this study, these predictions should be interpreted as supportive rather than definitive evidence of substrate specificity. Consequently, although DeepAden’s independent-probability design is, in principle, capable of representing substrate promiscuity, its current predictions should not be taken as definitive evidence of true biochemical multi-substrate usage and should be complemented by PCP-domain analysis, module-level context, and targeted biochemical assays. Rather, lower-ranked or lower-confidence entries within the top *k* predictions are best treated as hypotheses that may reflect latent pocket-level promiscuity, but still require corroboration from PCP-domain features, module- and pathway-level context, or direct experimental validation. In practice, users interested in potential promiscuity should therefore cautiously inspect top-*k* predictions rather than relying solely on a single top-1 assignment and interpret alternative substrates in light of structural and biochemical constraints downstream of the A-domain. To address these challenges, a promising future direction is to develop multimodal datasets that integrate atomic-level binding-pocket features and to design conformation feature extractors on the basis of geometric deep learning algorithms that enable “sequence-to-conformation-to-substrate” prediction [67, 68].

Furthermore, it is important to acknowledge the inherent limitations of substrate-prediction tools in the broader context of natural product discovery. While DeepAden shows competitive performance in predicting A-domain specificity on our benchmark datasets (Fig. 6), the translation of these predictions into full compound structures is complicated by noncanonical NRPS assembly logic. Several NRPS BGCs do not follow the standard colinearity rule. For instance, the biosynthesis of surugamides [69], wollamides [70], and desotamides [71] involves iterative mechanisms, trans-acting domains, or module skipping, which decouple the linear gene sequence from the final chemical structure. In such cases, accurate substrate prediction is only one piece of the puzzle. Therefore, while DeepAden provides useful substrate-level predictions for monomer incorporation, these results must be integrated with detailed biosynthetic-logic analysis to infer plausible natural product scaffolds rather than to claim fully resolved structures. In particular, the 27-AA ABP proposed in this study provides a compact and informative representation of the binding pocket that can support such integrative analyses. Given the performance of DeepAden in predicting nonproteinogenic substrates that exhibit high evolutionary divergence from GrsA-PheA, we anticipate that this tool will facilitate substrate-level functional annotation and prioritization of NRPS BGCs identified by genome mining, thereby providing useful support for natural-product-based drug discovery.

## Supplementary Material

gkag656_Supplemental_Files

## Data Availability

The source code is available at repository: https://doi.org/10.5281/zenodo.18754112. We also developed a website that allows users from any computer background to use it, which can be accessed at https://deepnp.site/. The web server usage and parameter settings were described in the Materials and methods section.
