# Supplementary material for: DeepAden: an explainable machine learning method for predicting the substrate specificity of nonribosomal peptide synthetases
Source: Nucleic Acids Res. 2026 Jul 6;54(13):gkag656. doi: 10.1093/nar/gkag656 (PMC13335487; doi:10.1093/nar/gkag656)
Supplement: gkag656_Supplemental_Files [file gkag656_supplemental_files.zip › 4. Huang_et_al_supplementary information_NAR-00630-Met-K-2026.pdf]

Supplementary data

DeepAden: An explainable machine learning method for predicting the substrate specificity of nonribosomal peptide synthetases

Jiaquan Huang[1,†], Liangjun Ge[1,†], Yaxin Wu[1,†], Yi Tian[1], Jun Wu[1], Qiandi Gao[1], Pan Li[1], Song Meng[2,3,4], Heqian Zhang[1,*], Zhiwei Qin[1,*]

[1]Center for Biological Science and Technology, Advanced Institute of Natural Sciences, Beijing Normal University, Zhuhai, Guangdong, 519087, China.

[2]State Key Laboratory of Drug Research & Natural Products Research Center, Shanghai Institute of Materia Medica, Chinese Academy of Sciences, Shanghai 201203, People's Republic of China.

[3]University of Chinese Academy of Sciences, Beijing 100049, People's Republic of China

[4]Zhongshan Institute for Drug Discovery, Shanghai Institute of Materia Medica, Chinese Academy of Sciences, Zhongshan, 528400, People's Republic of China.

*Correspondence: z.qin@bnu.edu.cn; zhangheqian@bnu.edu.cn

[†]These authors contributed equally to this work.

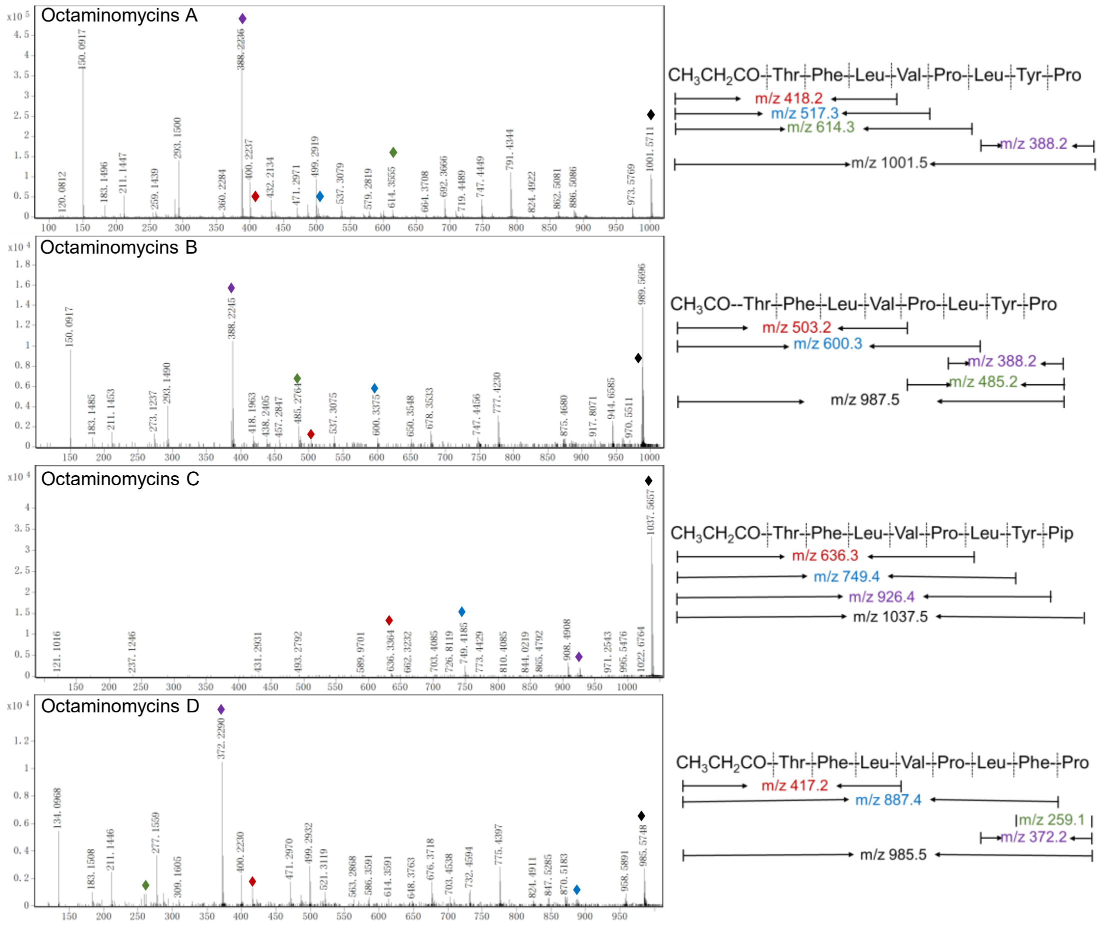

Supplementary Fig. S1. MS/MS spectra and diagnostic fragment ions used to establish the peptide backbones of octaminomycins A-D. (**Left**) Representative positive-mode MS/MS spectra of octaminomycins A-D, with the major sequence-informative ions highlighted. (**Right**) Summary of key fragment ions (color-coded by compound) whose m/z values correspond to consecutive N- or C-terminal peptide segments. These ions collectively confirm the proposed backbones and distinguish the four octaminomycin analogs from one another.

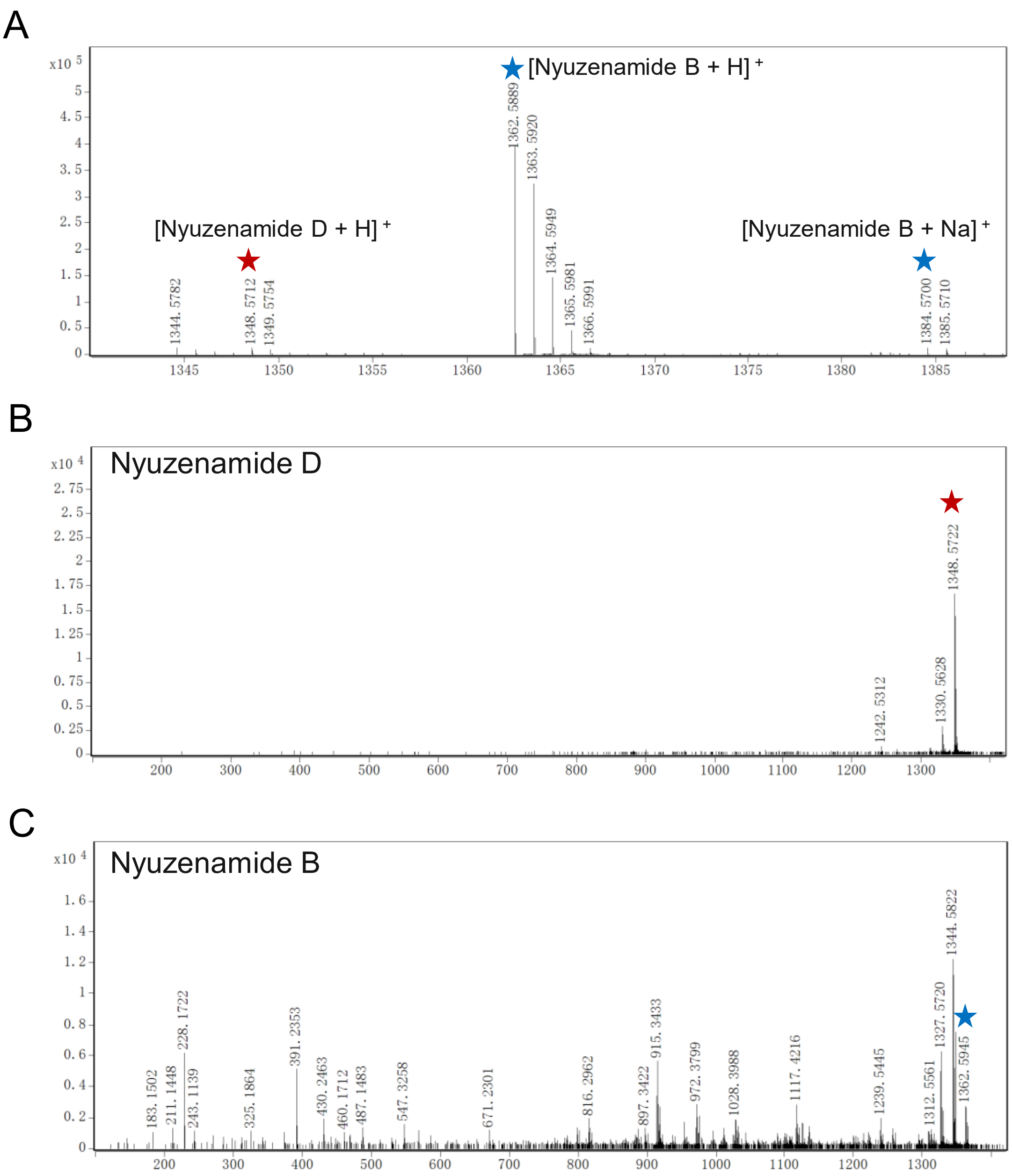

Supplementary Fig. S2. LC-MS and MS/MS analysis of nyuzenamides B and D from *S. hygroscopicus* OsiSh 2.

(A) High-resolution MS data showing the main ions assigned to nyuzenamides B and D, including [nyuzenamide B + H]$^+$, [nyuzenamide D + H]$^+$ and the sodium adduct [nyuzenamide B + Na]$^+$. **(B)** MS/MS spectrum of the nyuzenamide D precursor, which is dominated by precursor and neutral-loss ions with only limited backbone cleavage, consistent with its rigid bicyclic scaffold and stable ester linkages. The fragmentation pattern closely matches the GNPS reference spectrum of NMR-validated nyuzenamide D. **(C)** MS/MS spectrum of the nyuzenamide B precursor, showing a similar fragmentation behavior dominated by precursor and neutral-loss ions, in agreement with the reported nyuzenamide B structure.

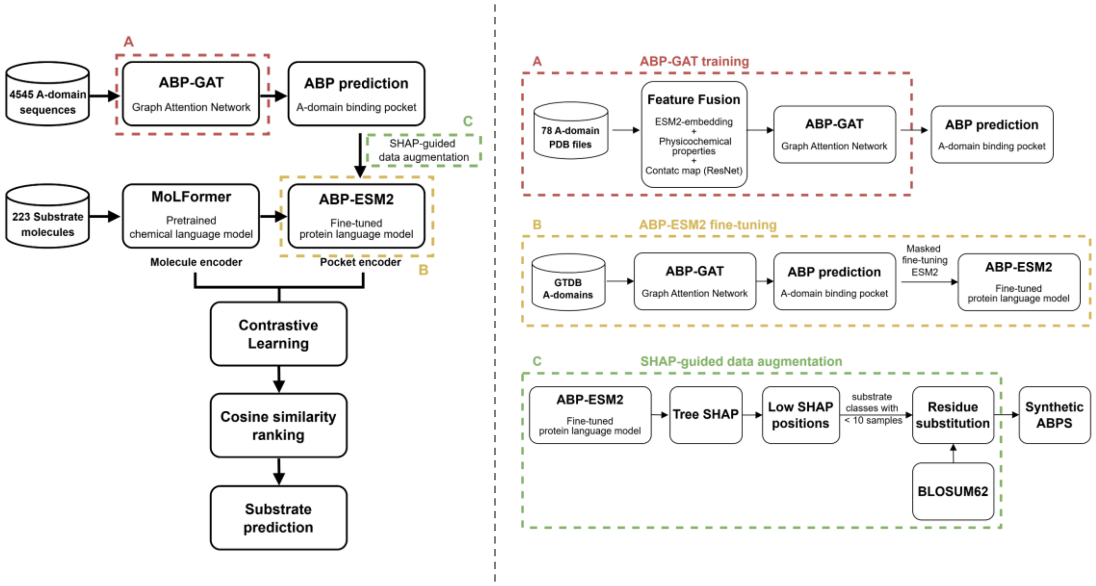

Supplementary Fig. S3. Overview of the DeepAden project framework. (Left) DeepAden integrates ABP-GAT for binding pocket prediction, ABP-ESM2 for pocket representation, and MoLFormer for substrate encoding within a contrastive learning framework for substrate prediction via cosine similarity ranking. (Right) Three core modules: (**A**) ABP-GAT training using fused structural and sequence features from 78 A-domain PDB structures; (**B**) ABP-ESM2 fine-tuning guided by ABP-GAT predictions on GTDB A-domains; (**C**) SHAP-guided data augmentation via BLOSUM62-based residue substitution at low-importance binding pocket positions to generate synthetic training data.

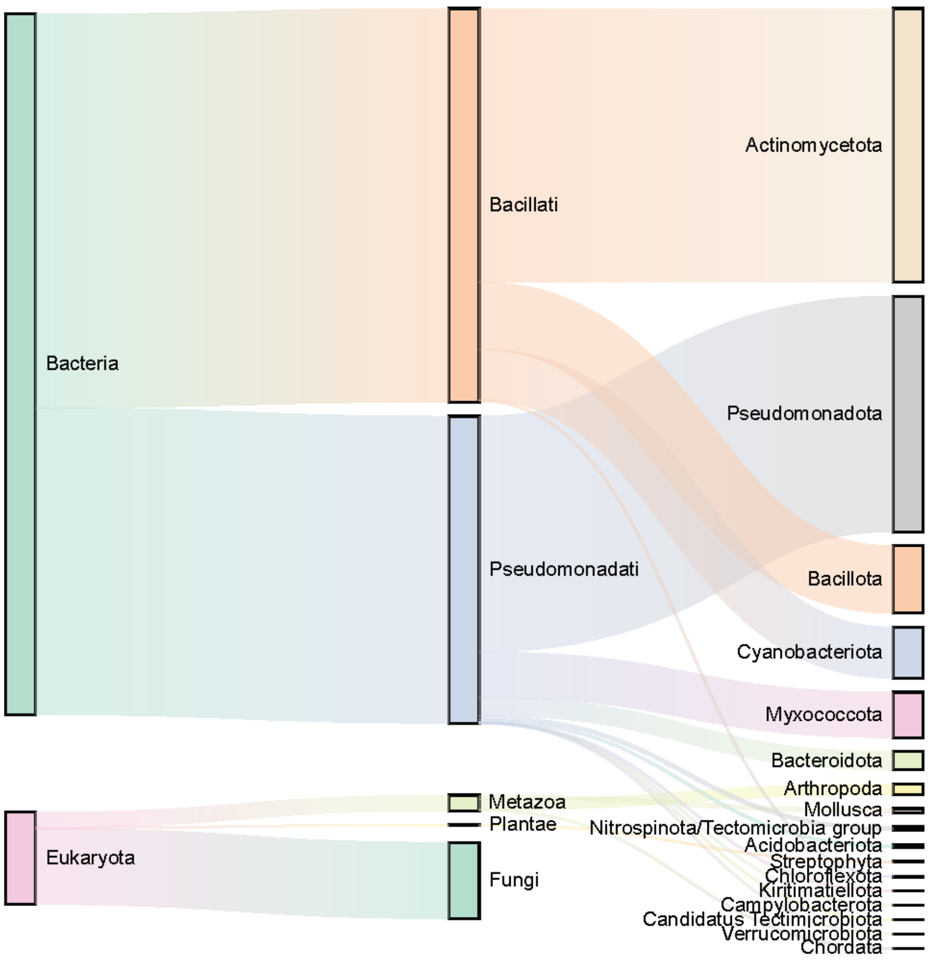

Supplementary Fig. S4. The 4545 A-domain organism sources of A-domain data used in our research.

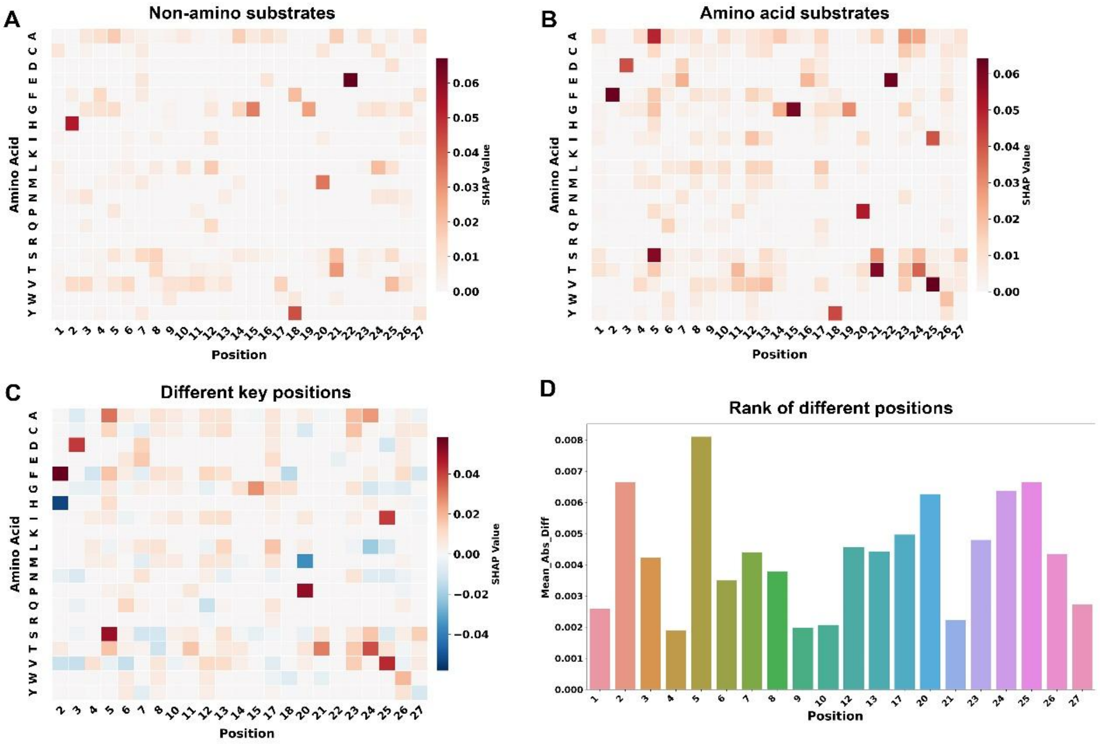

Supplementary Fig. S5. Heatmaps showing positive SHAP values that highlight amino acid contributions at each residue position for (**A**) non-amino ABPs (Dhb, PABA, Sal, Kiv, Cia, Pa, Ana, Ppa, 4-APCA, Qca, Box) and (**B**) amino acid substrate ABPs (Cys, Ser, Val, Gly, Pro, Ala, Thr, His, Asn, Gln, Tyr) within their respective classes. (**C**) Heatmap displaying the differences in key residue contributions between non-amino ABPs and amino acid substrate ABPs. (**D**) Bar plot ranking the SHAP value differences between non-amino ABPs and amino acid substrate ABPs.

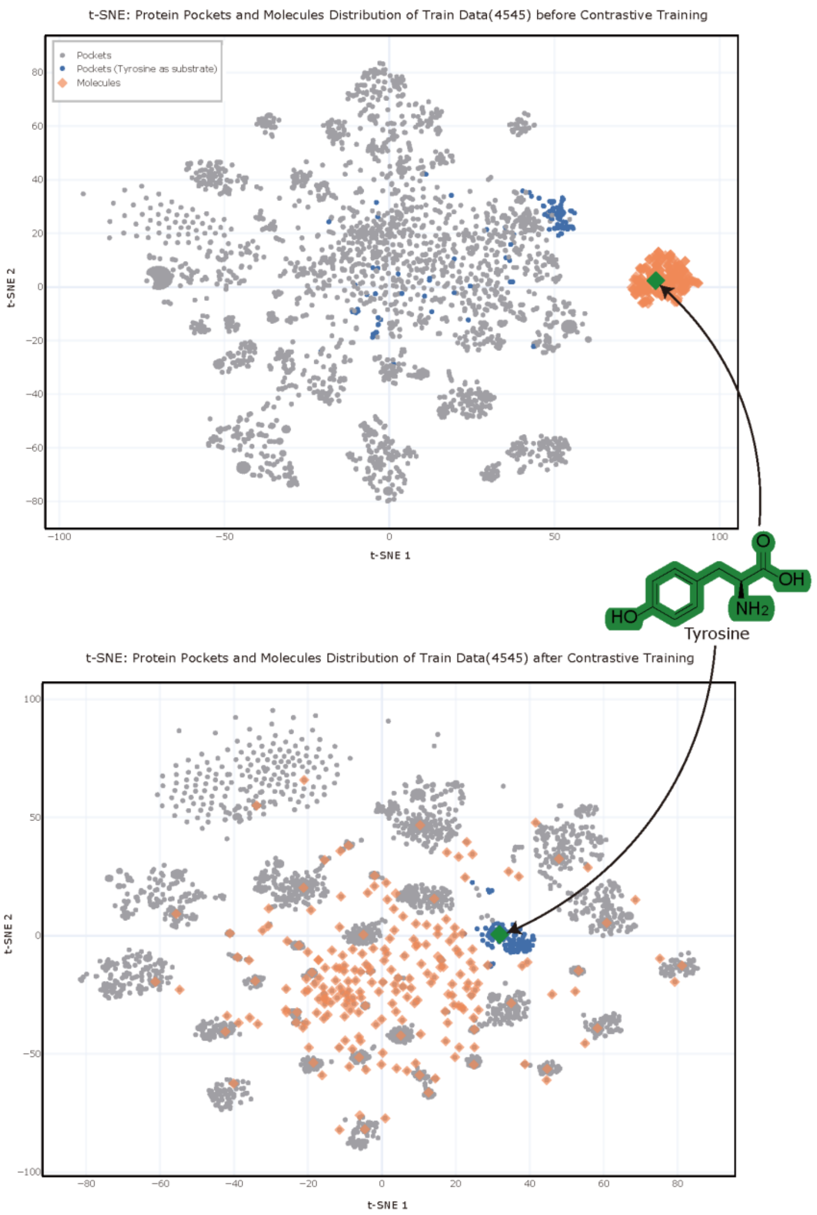

Supplementary Fig. S6. t-SNE visualization of 4545 A-doamin pocket sequences (dots) and their corresponding 223 substrate molecules (diamonds). Before contrastive training, the substrate molecule Tyrosine (green diamond) is distant from its corresponding pockets (blue dots) in the vector space. After contrastive training, Tyrosine and its corresponding pockets become closely clustered.

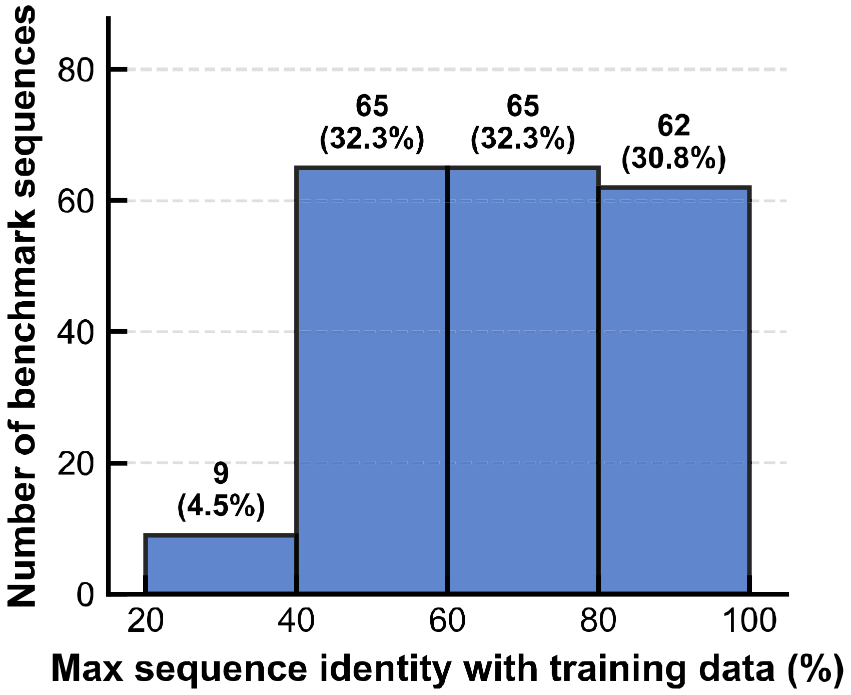

Supplementary Fig. S7. The histogram showing the maximum sequence identity between the 201-benchmark dataset and 4545 training datasets.

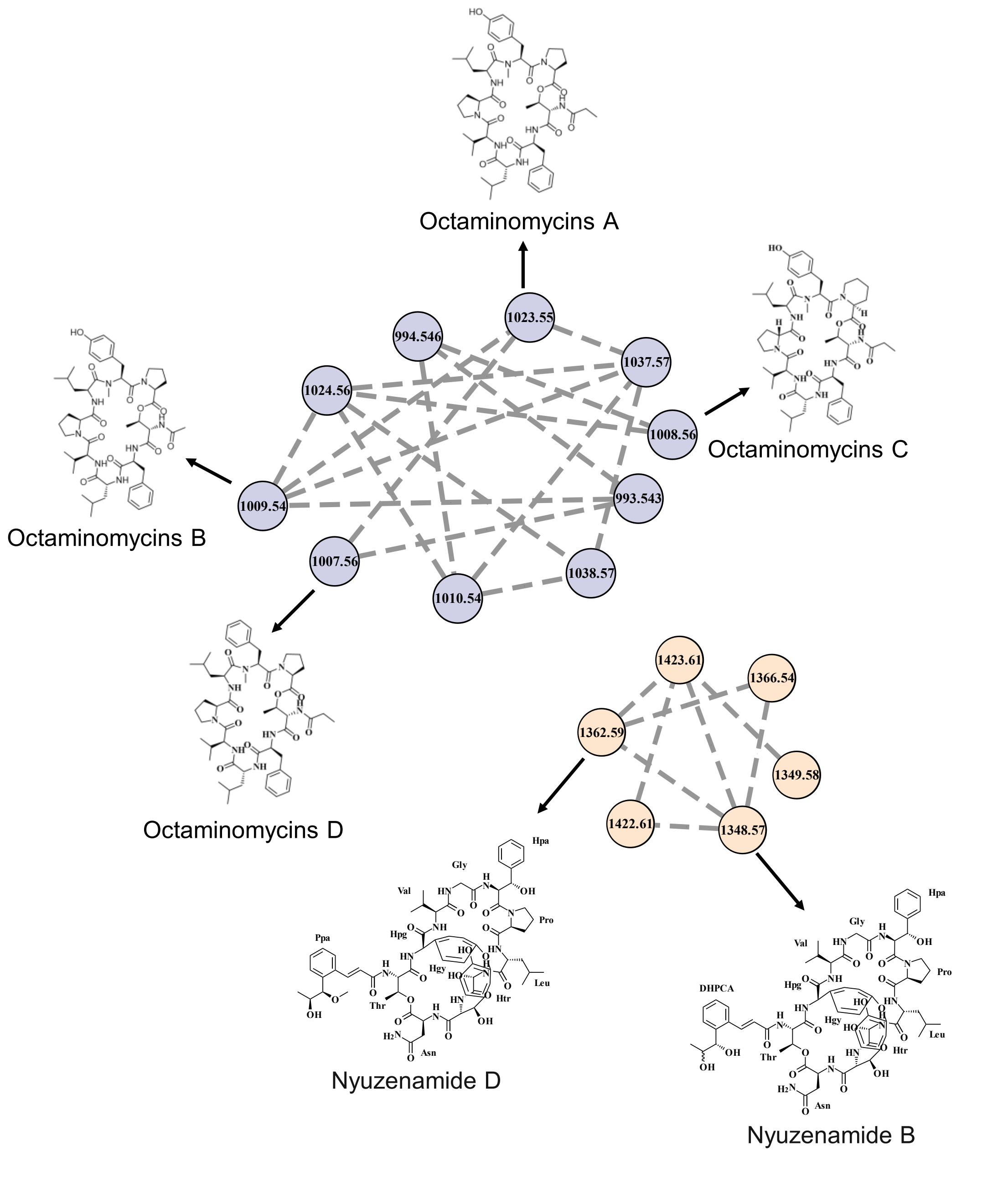

Supplementary Fig. S8. GNPS molecular networking of octaminomycins and nyuzenamide metabolites produced by *S. hygroscopicus* OsiSh 2. Nodes represent precursor ions (labeled by m/z), and edges indicate spectral similarity based on cosine scores. The purple cluster represents putative octaminomycins A–D with highly similar fragmentation patterns, consistent with the LC–MS and MS/MS data shown in **Fig. S1**. The orange cluster contains ions assigned to nyuzenamides B and D and their close analogues, consistent with the LC-MS and MS/MS data shown in **Fig. S2**.

Supplementary Table S1. Training parameters for each module in DeepAden

Model	Hyperparameters
Random forest-based A-domain binding pocket	n_estimators: 200<br>max_depth: None<br>min_samples_split: 2<br>min_samples_leaf: 1<br>bootstrap: False
Graph attention neural network-based A-domain binding pocket prediction model (ABP-GAT)	GAT model structure
	hidden_channels: 64<br>out_channels: 3<br>heads: 32<br>heads_intermediate: 16<br>heads_final: 8<br>dropratio: 0.5
	Training
	learning rate: 1e-3<br>epoch: 700<br>early_stop_epochs: 200<br>batch_size: 8
	Semi-supervised and data augmentation
	dropnode_rate: 0.4<br>dropedge_rate: 0.4<br>tem: 0.5<br>lam_initial: 0.1<br>lam_final: 0.5<br>lam_rampup_epochs: 300<br>order: 1
Multimodal contrastive learning-based pocket-substrate binding prediction model	epochs: 100<br>learning_rate: 1e-4<br>batch_size: 512<br>optimizer: adamw<br>weight_decay: 1e-4<br>temperature(τ): 0.1<br>early_stopping_patience: 10<br>early_stopping_delta: 1e-4

Supplementary Table S2. Construction of the 49 amino acids (49-AA) A-domain binding pocket within 8Å by comparing 10 co-crystal complexes of A-domains.

PDB ID	Region 1	Region 2	Region 3	Region 4	Region 5	substrate	Organism
**1amu**	---------L--<br>FF-----	A---FDASVWE--M	-TL--	-ITAGS	-NAYGPTETTICATT	phe	*Brevibacillus brevis*
**1md9**	--R----Y--S--<br>-S----	L---HNYPLSSPG-	-ALV-	-QVGGA	-QVFGMAEGLVNYTR	dhb	*Bacillus subtilis*
**2vsq**	---------L---<br>------	SN-AFDAFTFDFYA	-FATT	-LFGGE	-NCYGPT--TVFATA	leu	*Bacillus subtilis*
**4d56**	---------F---<br>-----Y	V---FDVA-EE---	WSLPT	VIIGGE	INCYGPTEGTIAVSL	tyr	*Planktothrix agardhii*
**4zxi**	-------------<br>------	----FDI-------	-----	---GGE	--VYGPTETTVWSSA	gly	*Acinetobacter baumannii* AB307-0294
**5n9x**	-------------<br>------	H---FDFS-W----	-NQT-	-VFGGE	-NMYGITETTVHATF	thr	*Streptomyces* sp.
**7xbu**	---------L--<br>W------	A---FDPS-QQ---	-DLVT	-IIGGE	NTIYGPTEAAVNAT-	cap	*Saccharothrix mutabilis subsp. capreolus*
**5wm2**	I-R----Y--N--<br>-S----	L---HNFALACP--	-AVV-	-QVGGS	-QVFGMAEGLLNY--	sal	*Streptomyces gandocaensis*
**3vnr**	-------------<br>------	H---FDFSVWE---	-NQTP	-IFGGE	-NGYGITETTVFTTF	aba	*Streptomyces sp.*
**8gic**	---------L---<br>--W---	AP--FDASLFE---	-HLTA	-LTGGD	RHLYGPTETTLCATW	hpg	*Actinoplanes teichomyceticus*
**49 AA**	X-X----X-XX-<br>XXXX--X	XX-XXXXXXXXXXX	XXXXX	XXXXXX	XXXXXXXXXXXXXXX	-	-

Supplementary Table S3. Comparison of amino acid residue positions in the 10-AA and 34-AA codes with the A-domain binding pocket residues. All residue numbering is based on GrsA_Phe (PDB: 1amu). Residues highlighted in cyan shadow represent informative positions identified by Terlouw et al (1), while residues highlighted in yellow shadow represent contributory positions identified in this study.

34-AA	27-AA	10-AA
210		
213		
214		
230	**230**	
234	**234**	
235	**235**	235
**236**	**236**	236
237	237	
238		
**239**	239	239
	240	
243		
	277	
**278**	**278**	278
279	279	
	280	
299	299	299
300	300	
**301**	301	301
302	302	
303	303	
320		
321		
**322**	322	322
323	323	
324	324	
325	**325**	
326	326	
327	327	
328	328	
329	**329**	
330	330	330
331	331	331
332	**332**	
333		
334		
		517

Supplementary Table S4. Pairwise bootstrap comparisons corresponding to Fig. 6C-E.

Analysis	Comparison	Mean difference (pp)	95% CI lower	95% CI upper
Overall top-3	DeepAden vs NRPStransformer	2.49	1.49	3.49
Overall top-3	DeepAden vs PARAS	3.32	2.48	4.98
Overall top-3	NRPStransformer vs PARAS	0.83	0.00	1.49
Identity-stratified	DeepAden vs NRPStransformer	9.20	2.77	20.63
Identity-stratified	DeepAden vs PARAS	2.18	-3.58	9.05
Identity-stratified	NRPStransformer vs PARAS	-7.02	-24.44	6.51
Proteinogenic	DeepAden vs NRPStransformer	2.76	-5.92	12.69
Proteinogenic	DeepAden vs PARAS	4.12	-1.22	11.26
Proteinogenic	NRPStransformer vs PARAS	1.36	-9.63	11.64
Nonproteinogenic	DeepAden vs NRPStransformer	23.81	3.17	46.03
Nonproteinogenic	DeepAden vs PARAS	18.25	3.17	35.71
Nonproteinogenic	NRPStransformer vs PARAS	-5.56	-27.78	17.46

Supplementary Table S5. Analysis with antiSMASH 7.0 (2) identified 12 NRPS biosynthetic gene clusters in *S. hygroscopicus* OsiSh-2.

Region	Type	From	To	Similarity Confidence	Most similar known cluster	NPRS module counts
4.2	NRP-metallophore, NRPS, NRPS-like, betalactone, T1PKS, T3PKS	82,996	244,645	Medium	totopotensamides	9
4.5	NRPS-like	727,861	769,813	-	-	1
4.8	T1PKS, NRPS	1,142,250	1,351,916	High	nigericin	8
4.15	PKS-like, transAT-PKS, NRPS, NRPS-like, redox-cofactor	2,599,787	2,704,451	Medium	Largimycins	2
4.19	NRPS-like	3,603,149	3,645,995	High	echosides	1
4.21	T1PKS, NRPS	4,445,308	4,568,264	High	alchivemycins	1
4.24	NRPS	5,605,917	5,688,015	Low	skyllamycins	10
4.26	terpene, NRPS	6,224,379	6,285,460	Medium	ochronotic pigment	1
4.33	NRPS-like, deazapurine, NRPS	8,115,635	8,163,925	Low	toyocamycin	3
4.36	T1PKS, NRPS-like	8,609,704	8,689,423	Medium	geldanamycin	1
4.39	terpene-precursor, NRPS-like	9,030,811	9,085,953	-	-	1
4.41	NRPS, lanthipeptide-class-i	9,186,165	9,275,988	-	-	10

Supplementary references

1. Terlouw, B.R., Huang, C., Meijer, D., Cediel-Becerra, J.D.D., Rothe, M.L., Jenner, M., Zhou, S., Zhang, Y., Fage, C.D., Tsunematsu, Y. *et al.* (2025) PARAS: high-accuracy machine-learning of substrate specificities in nonribosomal peptide synthetases. *bioRxiv*, 2025.2001.2008.631717.

2. Blin, K., Shaw, S., Augustijn, H.E., Reitz, Z.L., Biermann, F., Alanjary, M., Fetter, A., Terlouw, B.R., Metcalf, W.W., Helfrich, E.J.N. *et al.* (2023) antiSMASH 7.0: new and improved predictions for detection, regulation, chemical structures and visualisation. *Nucleic Acids Res*, **51**, W46-W50.
